# Role of anatomical sites and correlated risk factors on the survival of orthodontic miniscrew implants: a systematic review and meta-analysis

**DOI:** 10.1186/s40510-018-0225-1

**Published:** 2018-09-24

**Authors:** Hisham Mohammed, Khaled Wafaie, Mumen Z. Rizk, Mohammed Almuzian, Rami Sosly, David R. Bearn

**Affiliations:** 1 School of Dentistry, University of Dundee, Dundee, UK; 2 Discipline of Orthodontics, Faculty of Dentistry, University of Sydney, Sydney, Australia

**Keywords:** Failure rate, Miniscrew, Mini-implant, Orthodontic anchorage devices, Systematic review, Meta-analysis

## Abstract

**Objectives:**

The aim of this review was to systematically evaluate the failure rates of miniscrews related to their specific insertion site and explore the insertion site dependent risk factors contributing to their failure.

**Search methods:**

An electronic search was conducted in the Cochrane Central Register of Controlled Trials (CENTRAL), Web of Knowledge, Scopus, MEDLINE and PubMed up to October 2017. A comprehensive manual search was also performed.

**Eligibility criteria:**

Randomised clinical trials and prospective non-randomised studies, reporting a minimum of 20 inserted miniscrews in a specific insertion site and reporting the miniscrews’ failure rate in that insertion site, were included.

**Data collection and analysis:**

Study selection, data extraction and quality assessment were performed independently by two reviewers. Studies were sub-grouped according to the insertion site, and the failure rates for every individual insertion site were analysed using a random-effects model with corresponding 95% confidence interval. Sensitivity analyses were performed in order to test the robustness of the reported results.

**Results:**

Overall, 61 studies were included in the quantitative synthesis. Palatal sites had failure rates of 1.3% (95% CI 0.3–6), 4.8% (95% CI 1.6–13.4) and 5.5% (95% CI 2.8–10.7) for the midpalatal, paramedian and parapalatal insertion sites, respectively. The failure rates for the maxillary buccal sites were 9.2% (95% CI 7.4–11.4), 9.7% (95% CI 5.1–17.6) and 16.4% (95% CI 4.9–42.5) for the interradicular miniscrews inserted between maxillary first molars and second premolars and between maxillary canines and lateral incisors, and those inserted in the zygomatic buttress respectively. The failure rates for the mandibular buccal insertion sites were 13.5% (95% CI 7.3–23.6) and 9.9% (95% CI 4.9–19.1) for the interradicular miniscrews inserted between mandibular first molars and second premolars and between mandibular canines and first premolars, respectively. The risk of failure increased when the miniscrews contacted the roots, with a risk ratio of 8.7 (95% CI 5.1–14.7).

**Conclusions:**

Orthodontic miniscrew implants provide acceptable success rates that vary among the explored insertion sites. Very low to low quality of evidence suggests that miniscrews inserted in midpalatal locations have a failure rate of 1.3% and those inserted in the zygomatic buttress have a failure rate of 16.4%. Moderate quality of evidence indicates that root contact significantly contributes to the failure of interradicular miniscrews placed between the first molars and second premolars. Results should be interpreted with caution due to methodological drawbacks in some of the included studies.

**Electronic supplementary material:**

The online version of this article (10.1186/s40510-018-0225-1) contains supplementary material, which is available to authorized users.

## Background

Anchorage reinforcement is a crucial aspect of orthodontic treatment. Many extra-oral and intra- oral appliances had been used to control tooth movement and provide anchorage. Headgear use is known to be associated with compliance and co-operation problems [1, 2] with additional risks of causing serious injuries raising safety issues [3]. On the other hand, non-compliance intra-oral appliances such as palatal or lingual arches overcome the co-operation issues widely associated with extra-oral devices yet are accompanied with limited effectiveness in anchorage reinforcement [4–6].

The introduction of orthodontic miniscrew implants (OMIs) in the past decade has had a major impact on orthodontic treatment and added a whole new scope for orthodontic practices. Their convenience, simplicity and superior performance compared with conventional methods have contributed to their wide acceptance [7]. Several systematic reviews had shown the overall failure rates for OMIs with further exploration of the potential factors contributing to their failure [8–11]. However, OMIs could be placed in various insertion sites, and every one of those has its potential anatomical advantages and limitations [12, 13]. The in-depth exploration of the failure rates related to each independent insertion site and the insertion sites’ related risk factors contributing to the failure of OMIs has not been investigated before in a systematic review.

The objective of this systematic review was to answer the question of where should OMIs be inserted, presenting the cumulative failure rates for each independent insertion site and exploring the insertion sites’ associated risk factors contributing to the failure of OMIs.

## Materials and methods

### Protocol registration

This meta-analysis was planned and reported accordingly with the preferred reporting for systematic reviews (PRISMA) [14]. The protocol was registered *a priori* as a dissertation thesis (Additional file 1). However, it is not available online.

### Criteria for included studies

• Participants: Patients having orthodontic treatment and requiring the insertion of OMIs with no restriction over the type of orthodontic appliance or the presenting age of the patients.

• Intervention and comparators: Any orthodontic treatment intervention involving the insertion of OMIs at a designated insertion site (interradicular between specific teeth, midpalatal, paramedian, parapalatal, retromolar area or zygomatic buttress).

• Outcome: Primary outcome was the failure rate related to the specific OMI insertion site demonstrated by mobility, infection, inflammation or other factors leading to the premature loss of the OMI for the predefined study period. Possible specific insertion sites’ related risk factors contributing to the failure of OMIs such as root contacts, side of insertion, proximity to vital structures and cortical bone thickness would be additionally investigated.

• Study design: Only human randomised clinical trials (RCTs) and prospective non-randomised studies were included. Studies outside this scope with narrative nature, retrospective design, case reports and other designs were excluded. Only studies reporting a minimum of 20 placed OMIs in a specific insertion site were considered for inclusion.

### Search strategy

A comprehensive search using a combination of controlled vocabulary and free text terms was designed to allocate published, ongoing and unpublished studies. Electronic database searching was performed for the Cochrane Central Register of Controlled Trials (CENTRAL), Web of Knowledge, Scopus, MEDLINE and PubMed up to October 2017.

Other bibliographic databases were also searched for ongoing and unpublished data. A manual search was also carried out in relevant orthodontic journals until October 2017 (Additional file 2: Table S1). Besides, reference lists of the included articles and other relevant systematic reviews were screened for additional literature.

There was no restriction in the search strategy with regard to date; however, with the potential difficulties encountered with translating multiple articles into English, it was decided to only include articles presenting with a full text in English; however, this exclusion criterion was applied following the primary search so as to avoid bias in the search protocol.

### Data extraction and analysis

After removing duplicate studies using an Endnote reference manager software, relevant articles were identified after reading their titles and abstracts. Afterwards, the full texts of the potential articles were assessed for eligibility by two reviewers. Data extraction of the included studies was carried out independently by two reviewers using a pre-piloted standardised data extraction form. Disagreements were solved through discussion with a third reviewer. The data extraction form included the study identification, design of the study, setting, type of OMI, OMI dimensions, number of failed OMIs in relation to their specific insertion site and to their side of insertion. Another form was prepared for studies reporting on the insertion site-specific risk factors causing OMI failure including the outcomes related to cortical bone thickness, the influence of root contact and maxillary sinus perforation.

### Risk of bias and quality assessment in individual studies

Two reviewers independently performed the quality assessment, and the level of agreement was measured using the Kappa statistic [15] with the potential disagreements solved by a third reviewer. The Cochrane collaboration’s tool, a domain-based tool, was used for the assessment of the potential risk of bias of the randomised clinical trials assessing seven domains: sequence generation, allocation concealment, blinding of participants and personnel, blinding outcome assessor, incomplete data forms, selective reporting and finally other forms of bias [16]. The quality of the prospective non-randomised clinical trials was assessed using the star system of the Newcastle-Ottawa scale (NOS) [17]. A maximum of one star could be awarded for each of the four domains in selection of the groups; a maximum of two stars could be awarded for the single domain denoting comparability of the groups and finally a maximum of a single star could be awarded for the four domains in ascertainment of the outcome of interest accounting for a total of nine awarded stars. Studies with less than six awarded stars were judged to be of low quality while studies with six or more stars were considered to have a high quality. Authors were contacted if there were missing data that needed clarification.

### Summary measures and synthesis of the results

For palatal, maxillary buccal and mandibular insertion sites, data were eligible for pooling if two or more studies reported the failure rate for a specific insertion site. For dichotomous data, failure rates were noted as events and demonstrated as event rates with their corresponding 95% confidence intervals. Event rates were synthesised using a logit transformed proportion. For dichotomous data concerning the risk factors, data were noted as events and expressed as risk ratios as an effect estimate with 95% confidence intervals in a pairwise forest plot. Forest plots were generated using OpenMeta-Analyst. A random-effects model as described by DerSimonian and Laird [18] was considered *a priori* for the pooled estimates as it takes into its consideration the possible existence of heterogeneity. Statistical heterogeneity was assessed using the *I*^2^ statistic. A 25%, 50% and 75% statistic accounts for low, moderate and high levels of heterogeneity respectively. Moreover, the 95% predictive intervals around the treatment effects were calculated, whenever three or more studies were aggregated, to incorporate existing heterogeneity and predict possible treatment effects in future study settings. Studies were sub-grouped according to the OMI insertion site. The quality of the resultant evidence was graded following the recommendations outlined in the Cochrane handbook for systematic reviews of interventions [12].

### Additional analyses

Publication bias was inspected using the generated funnel plots whenever there were more than 10 studies. Further, a systematic assessment using Begg and Mazumdar’s rank correlation [19] and Egger’s linear regression tests [20] were utilised. Although the visual inspection of the generated funnel plots has been widely used for assessing publication bias, it is highly based on the subjective opinion of the viewer and its reliability is questionable [21] justifying the additional use of other analyses. To investigate the robustness of this review, additional sensitivity tests were performed investigating the impact of removing each individual study on the overall outcome. Additionally, studies with a non-randomised design and those reporting a small number of inserted OMIs (< 100) were further excluded.

## Results

### Study selection and characteristics

**Fig. 1:**
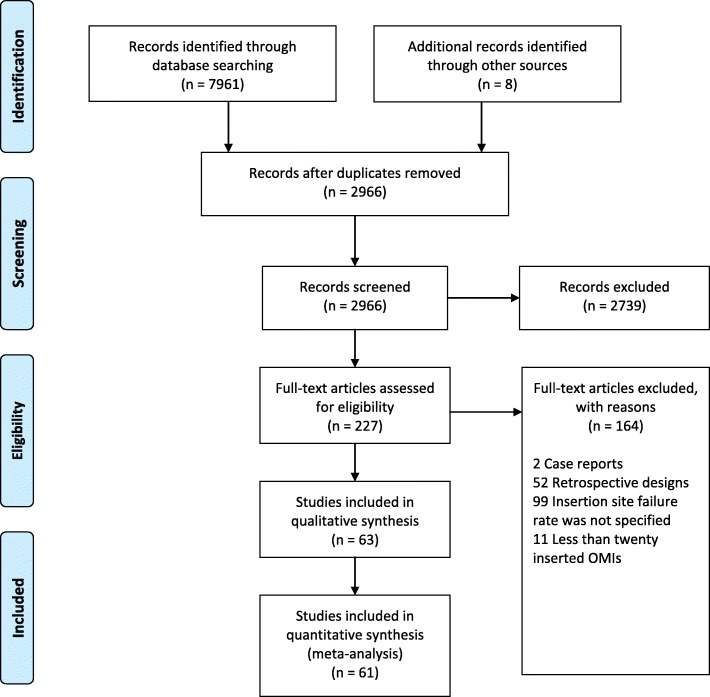
Diagram showing the study selection and identification

The initial electronic search yielded 7961 results supplemented by another 8 results obtained through the additional review of reference lists. 2966 studies remained after duplicate removal, and those were screened based on their titles and abstracts. A final sample of 227 articles with their full texts were retrieved and assessed against the inclusion/exclusion criteria leaving 63 articles after the exclusion of 164 articles (Fig. 1). The electronic database search with the applied search strategy and the list of articles excluded based on their full texts are provided (Additional file 2: Table S1).

Sixty-three studies met the inclusion criteria with 28 RCTs, 9 pCCTs and 26 pCS (Table 1). These studies reported on various insertion sites most notably in the interradicular areas between the first molars and second premolars and in palatal insertion sites. Authors were contacted whenever there were any unclear data that needed clarification. The full list of communications is presented in Additional file 3: Table S2. The additional reported risk factors including the impact of root contact, maxillary sinus penetration and bone density were gathered in a separate form (Additional file 4: Table S3).

**Table 1:** Characteristics of the included studies

Study	Design	Setting	OMI type	Length in (mm)	Diameter in (mm)	Specific location (failed/total)
Aboul-Ela [38]	RCT	University	AbsoAnchor, Dentos, Daegu, Korea	8	1.3	Mx first molar and second premolar (2/26)
Aglarci [39]	pCCT	NA	AbsoAnchor, Dentos, Daegu, Korea	10	1.6	Mx first molar and second premolar (6/50)
Akay [40]	pCS	University	Surgi-Tec	13	2.3	Parapalatal (0/40)
Al Maaitah [41]	pCCT	University	AbsoAnchor, Dentos, Daegu, Korea	8	1.3	Mx first molar and second premolar (5/44)
Al-Sibaie [42]	RCT	University	Dewimed, Tuttlingen, Germany	7	1.6	Mx first molar and second premolar (3/56)
Aras [43]	RCT	University	Anchor Plus, Los Angeles, Calif	6/7	1.4/1.6	-Mx first molar and second premolar (0/32) -Mx canine and lateral (1/32) -Right side (0/16); Left side (0/16)*
Aslan [44]	RCT	University	OrthoTechnology Inc., Tampa, Fla	8	1.5	-Mn canine and first premolar (4/32) -Right side (2/16); Left side (2/16)
Aydogdu [23]	RCT	University	AbsoAnchor, Dentos, Daegu, Korea	6	1.2	Mn lateral and canine (2/26)
Bechtold [45]	RCT	University	Orlus18107, Ortholution, Seoul, Korea	7	1.8	-Mx first molar and second premolar (3/24) -Mixed insertion sites (7/52)
Blaya [46]	pCS	Private	Sin Implant Systems, São Paulo, SP, Brazil	10	1.2	Mx first molar and second premolar (0/30)
Bushang [47]	pCS	University	IMTEC Corporation, Ardmore, OK	8	1.8	-Parapalatal (1/32) -Mn first molar and second premolar (1/22)
Canan [48]	RCT	University	Yesanchor, Seoul, Korea	9	1.8	Parapalatal (2/94)
Chen [49]	pCS	University	Ci Bei Corporation, Zhejiang, China	11	1.6	-Mx first molar and second premolar (2/40) -Mixed insertion sites (5/8)
Chopra [50]	RCT	Dental clinic	NA	NA	NA	-Mx first molar and second premolar (2/50) -Mn first molar and second premolar (3/50) -Right side (4/50); Left side (1/50)
Cozzani [51]	pCCT	NA	M.A.S., Micerium, Avegno, Italy	11	1.5	Paramedian (6/36)
Davoody [52]	RCT	University	NA	8/9	1.8/2	Mx first molar and second premolar (5/30)
Dawlatly [53]	RCT	University	OsteoCare™ Implant System, London, UK	9	1.8	Zygomatic buttress (1/20)
Duran [54]	pCS	Academy	Forestadent, Pforzheim, Germany	8	1.7	Paramedian (0/42)
Durrani [55]	RCT	University	NA	10	2	-Mx first molar and second premolar (10/60) -Right side (8/30); Left side (2/30)
Eissa [56]	RCT	University	MCT Tech, South Korea	10	1.6	-Mn canine and first premolar (0/30) -Right side (0/15); Left side (0/15)
El Beialy [57]	pCS	University	Absoanchor, Dentos, Daegu, Korea	8	1.2	-Mx first molar and second premolar (4/22) -Mn first molar and second premolar (3/18)
Elkordy [58]	RCT	University	3 M Unitek	10	1.6	-Mn canine and first premolar (3/30)
Ge [59]	RCT	University	ShenGang, ZhangHua, Taiwan	14	2	Zygomatic buttress (10/48)
Gelgor [60]	pCS	University	IMF Stryker, Leibinger, Germany	14	1.8	Midpalatal (0/25)
Gelgor [61]	pCCT	NA	IMF Stryker Leibinger, Germany	14	1.8	Midpalatal (0/40)
Gupta [62]	pCCT	University	Custom made Denticon, Mumbai	8	1.4	-Mx first molar and second premolar (9/40) -Right side (6/20); Left side (3/20)
Iwai [63]	RCT	University	ISA; Biodent, Tokyo, Japan	8	1.6	Mx first molar and second premolar (10/142)
Janson [64]	pCS	University	AbsoAnchor, Dentos, Daegu, Korea	7	1.5	Mx first molar and second premolar (4/40)
Kayalar [65]	RCT	University	Ortho Easy Forestadent	10	1.7	Paramedian (0/20)
Khan [66]	RCT	University	Dentaurum, Ispringen, Germany	8	1.2	-Mx first molar and second premolar (0/50) -Right side (0/25); Left side (0/25)
Kim [67]	pCS	University	C-implant, Seoul, Korea	8.5	1.8	-Mx first molar and second premolar (2/50) -Right side (1/25); Left side (1/25)
Lee [68]	pCCT	University	-Orlus18107, Ortholution, Seoul, Korea -Orthoplant; BioMaterials Korea Inc., Seoul, Korea	7	1.8/2.5	-Mx first molar and second premolar (2/36) -Mx first and second premolars (2/36)
Lehnen [69]	RCT	NA	Dentaurum, Ispringen, Germany	8	1.6	Mx first molar and second premolar (7/60)
Liou [32]	pCS	NA	Leibinger, Freiburg, Germany	17	2	-Zygomatic buttress (0/32) -Right side (0/16); Left side (0/16)
Liu [70]	RCT	NA	Cibei, Ningbo, China	8	1.2	Mx first molar and second premolar (8/68)
Manni [71]	RCT	NA	MAS, Micerium, Avegno, Italy	11	1.3–1.5	-Mn first molar and second premolar (0/50) -Right side (0/25); Left side (0/25)
Miresmaeili [72]	pCS	University	NA	8/10	1.4/1.6/2	Parapalatal (5/52)
Miyazawa [73]	pCS	University	Jeil Medical, Seoul, Korea	8	1.6	-Mx first molar and second premolar (3/23) -VL (1/21)
Motoyoshi [74]	pCS	University	ISA orthodontic Implant, BIODENT, Tokyo, Japan	8	1.6	-Mx first molar and second premolar (13/115) -Mn first molar and second premolar (11/94) -Right side (14/104); Left side (10/105)
Motoyoshi [75]	pCCT	University	ISA orthodontic Implant, BIODENT, Tokyo, Japan	8	1.6	Mx first molar and second premolar (7/143)
Motoyoshi [76]	pCS	University	ISA orthodontic Implant, BIODENT, Tokyo, Japan	8	1.6	-Mx first molar and second premolar (5/82) -Right side (3/41); Left side (2/41)
Motoyoshi [77]	pCS	University	ISA orthodontic Implant, BIODENT, Tokyo, Japan	8	1.6	-Mx first molar and second premolar (9/202) -Right side (6/102); Left side (3/100)
Nalçaci [78]	pCS	University	M-5146,11,Medartis AG, Basel, Switzerland	11	2	Paramedian (0/42)
Nienkemper [79]	pCS	University	PSM Medical Solutions, Tuttlingen, Germany	9	2	Paramedian (0/32)
Polat-Ozsoy [80]	pCS	University	AbsoAnchor, Dentos, Daegu, Korea	6	1.2	Mx lateral and canine (3/22)
Samrit [81]	pCS	NA	AbsoAnchor, Dentos, Daegu, Korea	NA	NA	-Mx first molar and second premolar (0/20) -Mn first molar and second premolar (4/18) -Right side (2/19); Left side (2/19)
Sarul [22]	RCT	University	Forestadent, Phorzheim, Germany	6/8	1.6	Mn first and second molars (14/54)
Saxena [82]	pCS	University	Custom made SK surgical	8	1.3	Mx lateral and canine (2/20)
Senisik [83]	RCT	University	Absoanchor; Dentos, Daegu, South Korea	5	1.3	Mx lateral and canine (3/30)
Sharma [84]	RCT	University	Denticon OMI	8	1.2	Mx first molar and second premolar (1/30)
Shigeeda [85]	pCS	University	ISA Orthodontic Mini-implants; Biodent, Tokyo, Japan	8	1.6	-Mx first molar and second premolar (3/79) -Mn first molar and second premolar (5/86)
Son [86]	RCT	University	ISA Orthodontic Biodent, Tokyo, Japan	8	1.6	-Mx first molar and second premolar (6/140) -Right side (5/70); Left side (1/70)
Suzuki [87]	pCS	University	-Sistema Nacional de Implantes, Sao Paulo, Brazil -ACR Mini-Implant, BioMaterials Korea, Guro-gu, Seoul, Korea	6/8	1.5	-Midpalatal (0/57) -Mixed insertion sites (19/223)
Suzuki [88]	RCT	University	AbsoAnchor; Dentos, Daegu, Korea	5/6/7	1.3	-Mx first molar and second premolar (8/122) -Mn first molar and second premolar (19/64) -Right side (16/93); Left side (11/93)
Toklu [89]	RCT	University	Total Anchor; Trimed, Ankara, Turkey	9	1.8	Paramedian (2/26)
Tuncer [90]	RCT	University	Absoanchor; Dentos, Daegu, Korea	7	1.4/1.5	-Mx first molar and second premolar (7/60)
Turkoz [91]	RCT	University	Absoanchor; Dentos, Daegu, Korea	7	1.4	Mx first molar and second premolar (25/112)
Upadhyay [92]	pCCT	University	Custom made OMI	8	1.3	Mx first molar and second premolar (4/30)
Upadhyay [93]	pCS	University	NA	8	1.3	Mx first molar and second premolar (2/46)
Viwattanatipa [94]	pCS	University	Osteomed, Dallas, Tex	8/10/12	1.2	-Mx first molar and second premolar (7/44) -Zygomatic buttress (25/53)
Von Bremen [95]	pCCT	Private	OrthoEasy®, Forestadent, Germany	8	1.8	-Mn first molar and second premolar (10/34) -Right side (5/17); Left side (5/17)
Watanabe [96]	pCS	University	Absoanchor Dentos Inc., Taegu, Korea	5/6/8	1.4	-Mx first molar and second premolar (11/132) -Mn first molar and second premolar (17/58)
Watanabe [97]	pCS	University	Dual-top Auto Screw III; Jeil Medical, Seoul, Korea	6	1.4	-Mx first molar and second premolar (11/50) -Parapalatal (6/70)

*RCT* randomised clinical trial, *pCCT* prospective controlled clinical trial, *pCS* prospective cohort study, *VL* various locations, *Mn* mandibular interradicular, *Mx* maxillary interradicular, *NA* not available

*Side of insertion data were only presented for miniscrews inserted between the maxillary first molar and second premolar

### Risk of bias within studies

**Fig. 2:**
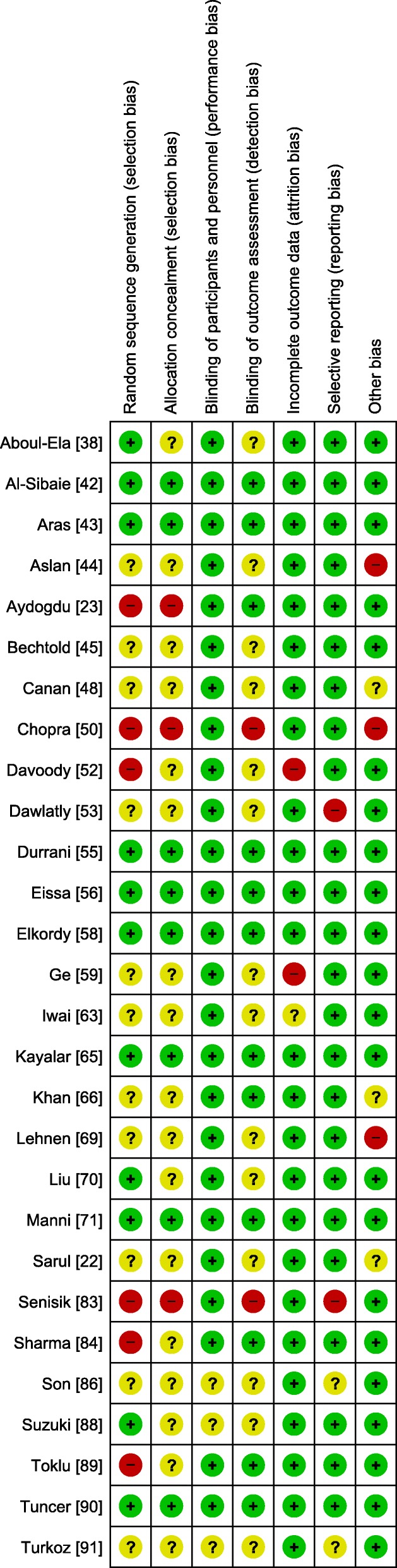
Risk of bias summary, high risk of bias (red), low risk of bias (green), and unclear risk of bias (yellow)

**Fig. 3:**
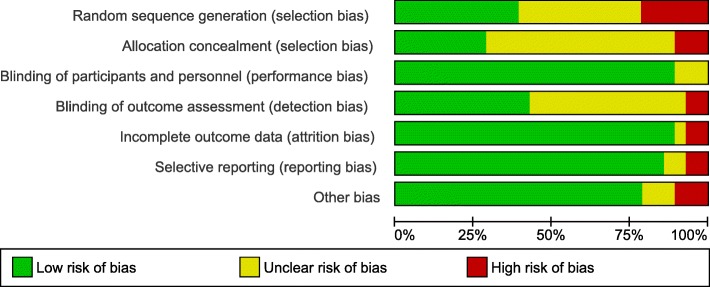
Risk of bias graph

Based on the overall score within all of the assessed domains, eight RCTs had an overall low risk of bias. Ten studies had an overall high risk of bias, and the rest had an unclear risk of bias. The risk of bias within the included studies is presented (Figs. 2 and 3).

**Fig. 4:**
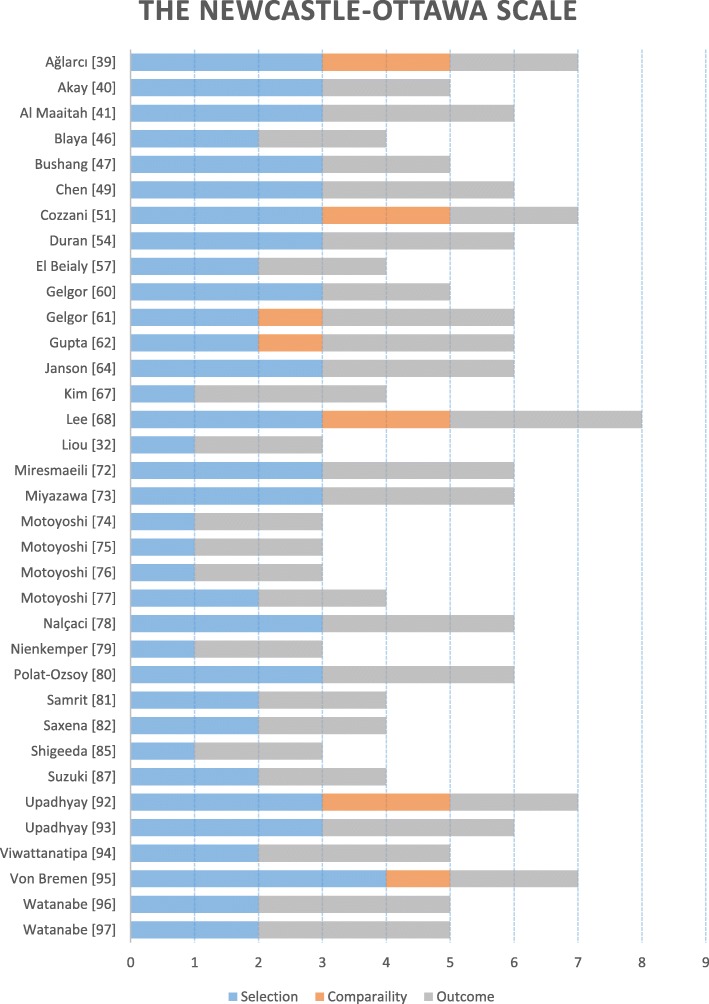
The Newcastle-Ottawa scale for the assessment of the quality of the prospective non-randomised studies

Sixteen prospective non-randomised studies scored six or more points on the NOS star grading system and were assessed to be of high quality. The other nineteen studies were judged to have a low overall quality scoring less than six points (Fig. 4).

The Kappa statistic between the two examiners was found to be 0.91 indicating a good level of agreement between the examiners [15].

### Results of individual studies and synthesis of the results

#### Palatal insertion sites

**Fig. 5:**
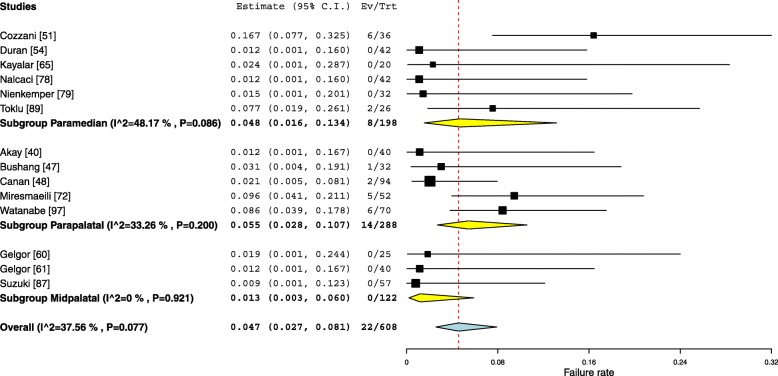
Forest plot showing the failure rates for various palatal insertion sites

Three palatal insertion sites (Midpalatal, Paramedian and Parapalatal) were investigated in this systematic review. Overall, the failure for the OMIs inserted in the palate was found to be 4.7% (95% Cl 2.7–8.1). The failure rates of the OMIs inserted in the midpalatal region, in three studies, were pooled together to produce an overall failure of (1.3%, 95% Cl 0.3–6). However, the aggregated figures, from six studies that tested the failure rate of OMIs inserted in the paramedian region, was 4.8% (95% Cl 1.6–13.4). Similarly, the failure rates of the OMIs inserted in the parapalatal area were aggregated from five studies to produce an overall 5.5% (95% Cl 2.8–10.7) failure rate (Fig. 5).

#### Maxillary buccal insertion sites

**Fig. 6:**
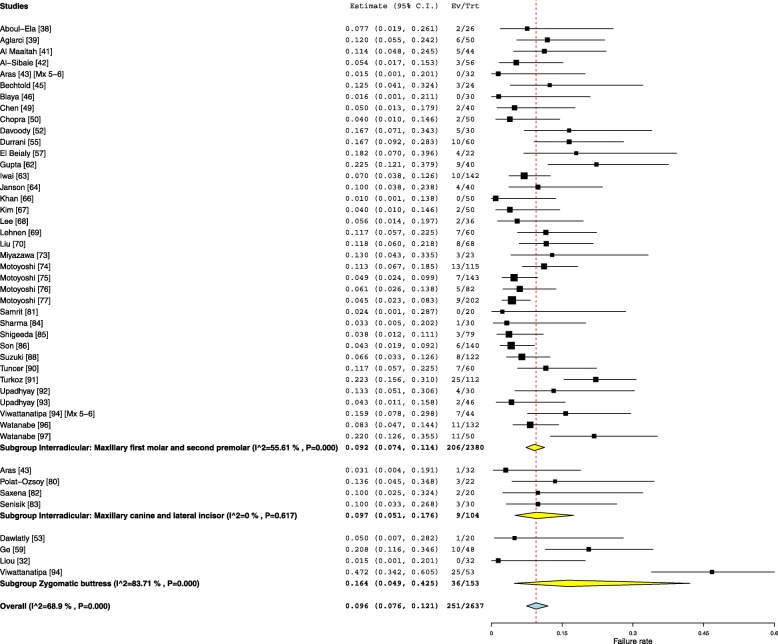
Forest plot showing the failure rates for various maxillary buccal insertion sites

Three maxillary buccal OMI locations were analysed in this review. The overall failure for the maxillary buccal insertion sites was found to be 9.6% (95% Cl 7.6–12.1). Thirty-seven studies explored the failure rate for OMIs placed in the interradicular area between the maxillary first molar and second premolar with an overall 9.2% (95% Cl 7.4–11.4) failure rate. The aggregated failure rates of the OMIs inserted between the maxillary lateral incisor and canine were pooled to produce an overall 9.7% (95% Cl 5.1–17.6) failure rate, while the aggregated failure rates of the OMIs inserted in the zygomatic buttress location from four studies was found to be 16.4% (95% Cl 4.9–42.5) (Fig. 6).

#### Mandibular insertion sites

**Fig. 7:**
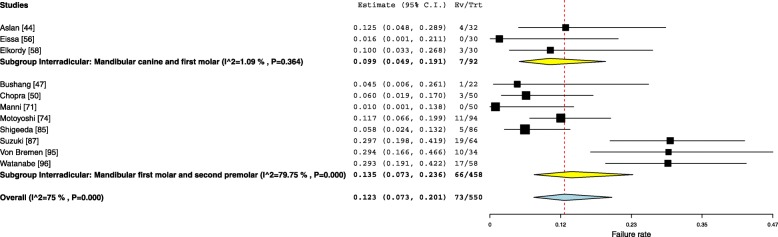
Forest plot showing the failure rates for various mandibular buccal insertion sites

Two mandibular insertion sites were included in the quantitative synthesis which showed an overall failure rate of 12.3% (95% Cl 7.3–20.1). Eight studies reported on the failure rates for OMIs inserted between the mandibular first molar and second premolar with an overall 13.5% (95% Cl 7.3–23.6) failure rate. Three studies were aggregated for the interradicular insertion of the OMIs between the mandibular canine and first premolar, resulting in a 9.9% (95% Cl 4.9–19.1) failure rate (Fig. 7). One study investigated the failure rate for OMIs inserted in the interradicular location between the mandibular first and second molar and reported a 25.9% failure rate [22]. Another study reported a 7.6% failure rate for OMIs placed between the mandibular canine and lateral incisor [23]. These two studies [22, 23] were not included in the quantitative analysis.

### Risk factors

**Fig. 8:**
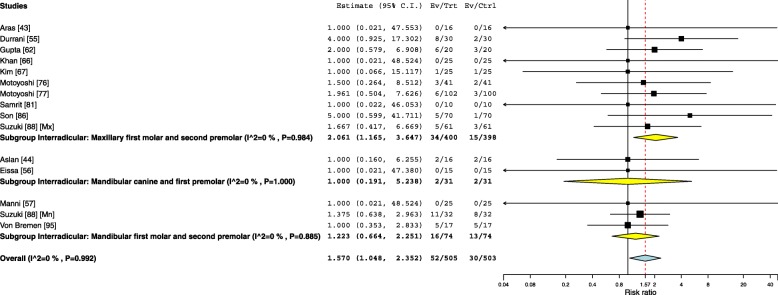
Forest plot showing the risk ratio for failure when OMIs where placed in the right versus the left sides

**Fig. 9:**
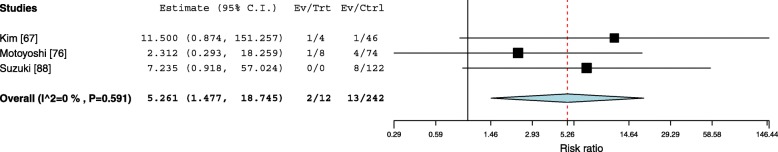
Forest plot showing the risk ratio for failure when OMIs penetrated the maxillary sinus. Trt (OMIs perforating sinus), Ctrl (OMIs without sinus perforation)

**Fig. 10:**
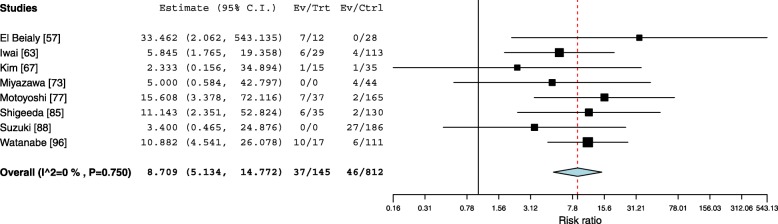
Forest plot showing the risk ratio for failure when OMIs contacted the roots, Trt (root contact), Ctrl (no root contact)

Fourteen studies investigated the influence of side of insertion on interradicular OMI failure with an overall pooled risk ratio of 1.57 (95% CI 1.04–2.35) leaning towards more failures in the right side. The results were significant when the OMIs were inserted between the maxillary first molars and second premolars with a risk ratio of 2.06 (95% CI 1.16–3.64). On the other hand, a statistically non-significant risk ratio of 1.000 (95% CI 0.191–5.238) and 1.2 (95% CI 0.6–2.2) was observed when the OMIs were placed between the mandibular canine and first premolar and between the mandibular first molar and second premolar respectively (Fig. 8). The influence of maxillary sinus penetration on the failure rates of OMIs inserted between the maxillary first molars and second premolars was investigated in three studies with a statistically significant risk ratio of 5.26 (95% CI 1.47–18.74) (Fig. 9). Eight studies investigated the impact of root contact on OMI failure for OMIs inserted between the first molars and second premolars and assessed with cone beam tomography ending with a pooled risk ratio of 8.7 (95% Cl 5.1–14.7) (Fig. 10).

### Assessment of publication bias and grading of resultant evidence

**Fig. 11:**
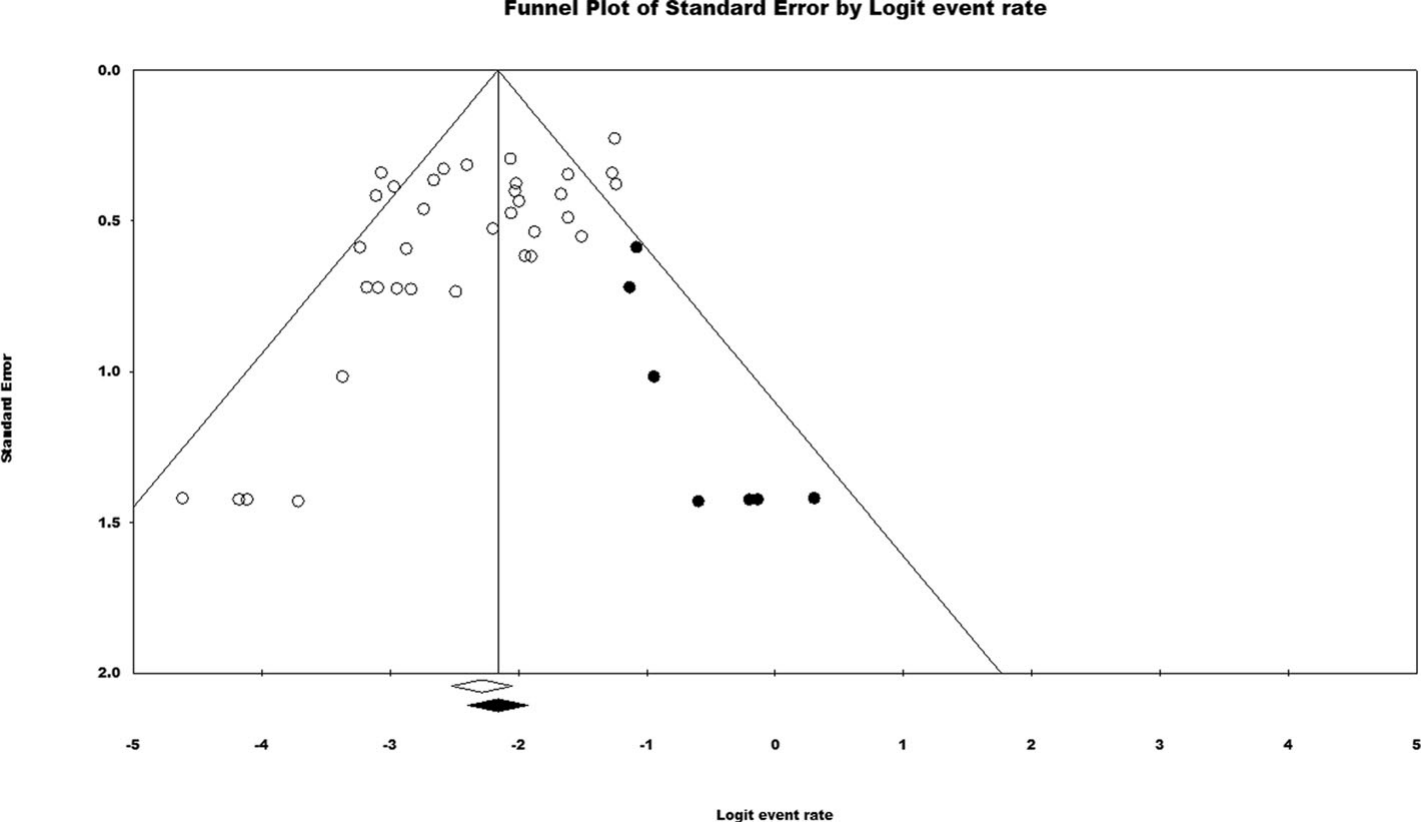
Funnel plot of the studies reporting on the interradicular location between the maxillary first molar and second premolar (black dots denote the imputed missing studies)

The inspection of the funnel plot (Fig. 11) that was generated for the pooled studies investigating the OMIs’ placement between the maxillary first molar and second premolar showed an asymmetrical pattern in the bottom of the funnel plot. Egger’s test reported a (*p* value < 0.001) with Begg and Mazumdar’s test having a value of 0.02 which hints towards the existence of publication bias [24]. An adjusted effect estimate of 10.9% (95% CI 9.6–12.4) was imputed following the trim and fill method to gauge the effect of those missing studies.

The quality of the resultant evidence ranged from very low to low mainly due to the design and limitations observed within some of the included studies. However, the quality of evidence was found to be moderate for the impact of root contact on the failure of OMIs. A detailed explanation of each outcome is represented in Additional file 5: Table S4.

### Sensitivity analyses

Sensitivity analysis was performed to investigate the robustness of the results by assessing the impact of each individual study on the overall results and the inclusion of non-randomised studies. No significant differences were noted when individual or small studies were excluded from the original synthesis. On the other hand, exclusion of non-randomised studies resulted in a modest decrease in the failure rates when OMIs are placed in palatal or maxillary buccal insertion sites. However, failure rates were significantly decreased for OMIs inserted between the mandibular first molars and second premolars when non-randomised studies were excluded 8.4% (95% CI 1.4–37.8). Exclusion of non-randomised studies investigating the influence of maxillary sinus perforation on OMI failure was not carried out due to the presence of only one RCT **(**Additional file 6: Figure S1.).

Considerable heterogeneity was observed for the pooled results from the zygomatic buttress with an *I*^2^ value of 83.7 and 79.75% for the OMIs inserted between the mandibular first molar and second premolar. Sources of heterogeneity were further gauged by the additional analyses that were performed. However, other possible variables like operators’ experience, surgical placement techniques and others were not adequately reported within the included studies and should be thoroughly addressed in future study settings. A detailed summary of the results obtained from the quantitative synthesis and sensitivity analyses are presented in (Additional file 7: Table S5).

## Discussion

### Summary of evidence

**Fig. 12:**
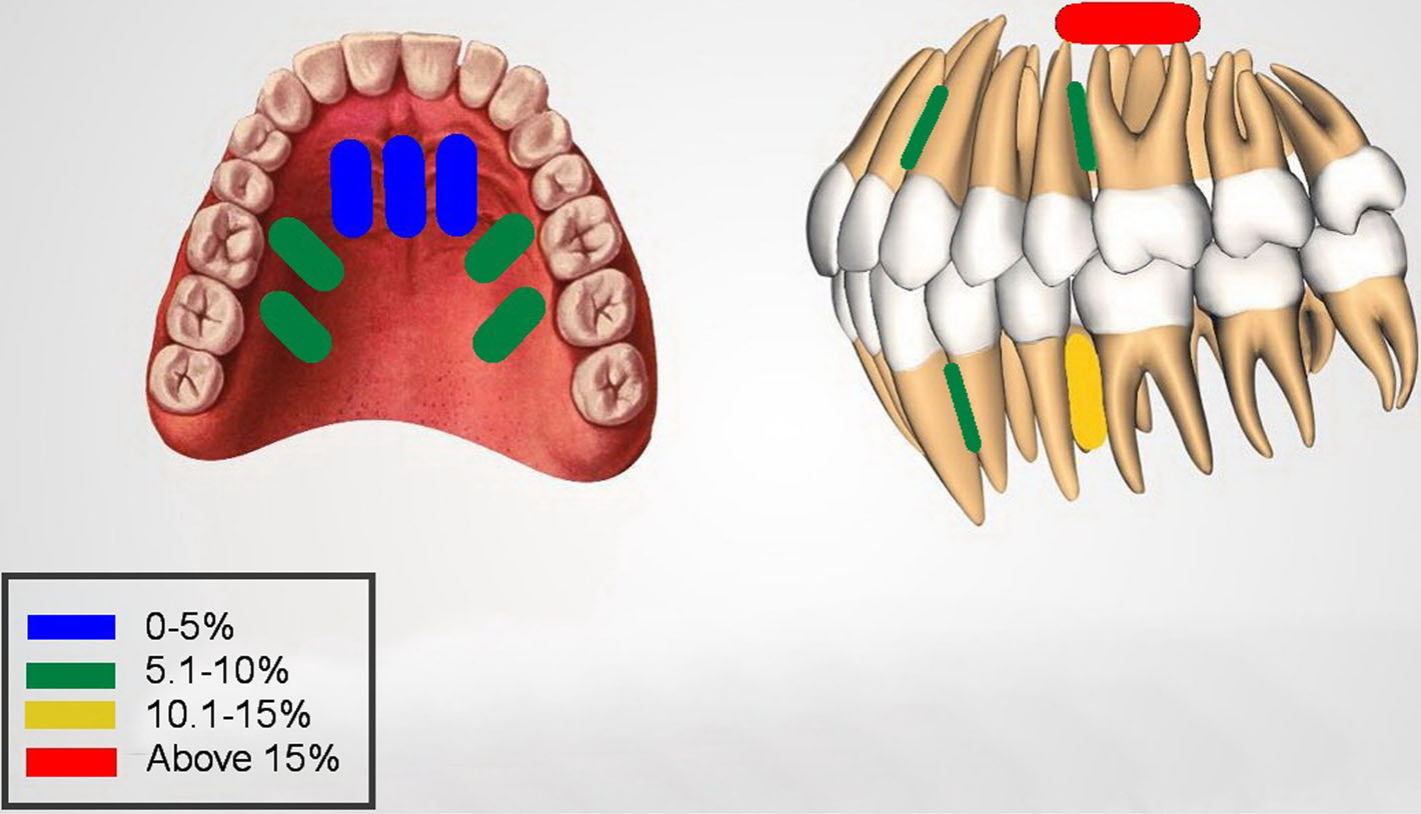
Diagram demonstrating failure rates obtained from the quantitative synthesis

There is no doubt that the quality of healthcare delivery to potential orthodontic patients is affected by the selection of the OMI insertion site. A problematic location might result in hindering the efficacy of orthodontic treatment through consistent failures, thus, prolonging treatment time. It is important to understand the anatomical limitations of various OMI insertion sites. This review attempts to provide a clinical guide to the selection of the appropriate OMI insertion sites as well as present an insight into the risk factors that might potentially influence OMI failure. A summarised diagrammatic presentation of the analysed OMIs locations, based on the quantitative synthesis, is shown in Fig. 12.

### Palatal sites

The palate naturally offers an excellent location for OMI insertion being far from the roots of the teeth. The midpalatal area had the lowest failure rate (1.3%) offering an excellent location for OMI insertion. This might be attributed to the thinness of the soft tissues and the quality of the cortical bone in this area, altogether with the direct ease of insertion [25, 26]. The failure rate for the paramedian OMIs was 4.8% falling just behind the midpalatal area and demonstrating another excellent alternative for OMI insertion. The parapalatal area is utilised by inserting OMIs in the lateral borders of the palate. This insertion site has been widely used for intrusion purposes of the maxillary posterior teeth [27, 28]. The failure rate for the parapalatal area was 5.5% acting as a good alternative palatal insertion site. When compared to the other insertion sites, the possibility of touching the roots in the parapalatal is higher and that might be the reason for the increased failure rate in this palatal OMI anatomical insertion site.

### Maxillary buccal insertion sites

The most popular insertion site for the OMIs is between the maxillary first molars and second premolars with thirty seven studies being aggregated to produce a total of 9.2% failure rate in this insertion site. This location provides simple and, yet, effective mechanics for handling premolar extraction cases, hence, showing acceptable success rates. The insertion site between the maxillary canine and the lateral incisor had an almost identical failure rate of 9.7%, both falling behind the palatal sites in terms of success rates. This can be attributed to the closeness of the roots to OMIs insertion sites. Another popular interradicular insertion site for the intrusion of anterior teeth is between the maxillary centrals. However, the studies reporting on this subject had small sample sizes and were not included in this review [29–31]. The least successful insertion site was the zygomatic buttress, the pillar of the cortical bone along the zygomatic process in the maxilla [32], with an overall failure rate of 16.4%, the highest among all of the investigated insertion sites. This high failure rate might be explained by the nature of the movable gingiva at the insertion site of the OMIs as well as the poor accessibility to this site during insertion and cleaning. Another aspect observed in this review was the significant heterogeneity among the included studies in terms of the failure rate of OMIs inserted in the maxillary buccal regions; this may be due to the different methodologies (different insertion techniques, i.e. surgical and non-surgical placement).

### Mandibular insertion sites

The most common insertion site in the mandible is between the mandibular first molars and second premolars with a reported failure rate of 13.5%. This higher failure rate is consistent with the findings of previous systematic reviews [9, 10, 33]. Another popular mandibular insertion site is between the roots of the first premolars and canines. OMIs inserted in this region are commonly utilised for mesialising the posterior mandibular dentition and as a method of anchorage reinforcement with fixed functional appliances. This review showed that OMIs inserted between the roots of the first premolars and canines have a failure rate just below 10%.

On the other hand, one study showed that OMIs inserted between the mandibular molars have a high failure rate (26%) [22]. This could be attributed to two reasons, first, the moveable gingivae covering the region of insertion and, secondly, the difficult access. A single study reported that four out of a total of 26 OMIs inserted between the mandibular canine and lateral incisor showed some degree of mobility; however, two of them were capable of still serving their function [23]. This low success rate of the inserted OMIs could be associated with the thin mobilised and non-keratinised gingivae at the region of insertion.

### Risk factors

Multiple factors influence the success of OMIs which subsequently influence the end results reported by different studies [34, 35]. Insertion sites’ related risk factors are the risk factors presenting limitations to a selected insertion site. Those factors could be due to anatomical limitations such as the proximity to vital structures and roots, soft tissue thickness, cortical bone density/quality or the side of insertion. Root contact and maxillary sinus perforation were found to have a significant contribution to the failure of OMIs inserted between the first molars and second premolars. These results manifest in a clinically significant effect explaining the less successful outcomes between the interradicular OMIs and those inserted in the palate. This indicates that avoiding root contact when utilizing an interradicular insertion site is paramount [36, 37]. The right side has been reported in many studies to have higher failure rates which might be attributed to the operator’s hand preference altogether with the patient’s brushing preference. However, in our review, the results were marginally significant in favour of the left side which is less likely to produce an impact on the clinical decisions. On the other hand, the differences in the methods of analysis, units of analysis and insertion locations in studies investigating the influence of bone densities hindered the aggregation of these results into a quantitative synthesis.

### Overall limitations

The included studies were only in the English language. A decision was made to only include the English articles due to the difficulty and limited resources in obtaining translations of large number of articles in a variety of different languages.

Studies with less than 20 inserted OMIs were excluded. Although this could be considered as a limitation, these studies probably lack the power to influence the outcomes of data synthesis.

The authors acknowledge that many studies presented in this review have shown poor methodological design with wide confidence intervals within some of the generated quantitative analyses; this in turn decreases the conclusiveness of the presented findings.

### Recommendations for clinical practice

The results presented in this review provide a useful clinical guide for the selection of appropriate insertion sites for OMIs. Based on the available evidence, high success rates can be expected from the OMIs placed in the palate whether they are placed in the midpalatal, paramedian or the parapalatal areas with no notable reported adverse effects. The success rates of OMIs placed in the interradicular maxillary areas, between the first molars and second premolars and between the lateral incisors and canines were found to be acceptable altogether with those placed in the mandible between the canine and first premolar. OMIs placed in the mandible between the first molars and second premolars and those inserted in the zygomatic buttress showed the highest failure rates, though they can still be used as their success rates may still be considered within the acceptable limits. The choice of OMI insertion site falls on the clinician, but patients should be informed about the potential failure rates related to the choice of site and the possible associated risk factors.

### Recommendations for future research

Although this review included 63 studies, some insertion sites were not investigated and others were only reported in a small number of trials. Another problem presented itself as many studies suffered methodological drawbacks. High-quality RCTs, investigating this crucial area and exploring various insertion sites, are needed. Ideally, trials should include a proper sequence generation, allocation concealment, blinding, reporting of the results and adequately powered through a prior sample size calculation.

## Conclusions

- Orthodontic miniscrew implants provide acceptable success rates that vary among the explored insertion sites.
- Low quality of evidence suggests that miniscrew implants inserted in palatal locations exhibit a failure rate of 1.3, 4.8 and 5.5% for the midpalatal, paramedian and parapalatal locations respectively.
- Very low quality of evidence suggests that miniscrews inserted in the zygomatic buttress suffer from a failure rate of 16.4%.
- Very low quality of evidence also suggests that miniscrews inserted in interradicular locations between the first molars and second premolars suffer from a failure rate of 9.2% for those inserted in the maxilla and 13.5% for those inserted in the mandible.
- Moderate quality of evidence indicates that root contact is a major risk factor contributing to the failure of orthodontic miniscrew implants inserted between the first molars and second premolars.
- Further high-quality research is needed to investigate other commonly utilised insertion sites while reporting on the different possible risk factors.

## Additional files

Additional file 1: Protocol. (PDF 185 kb)

Additional file 2: **Table S1**. Databases, search strategies and exclusions. (PDF 271 kb)

Additional file 3: **Table S2**. List of communications. (PDF 137 kb)

Additional file 4: **Table S3**. Additional risk factors. (PDF 168 kb)

Additional file 5: **Table S4**. Grading of evidence. (PDF 848 kb)

Additional file 6: **Figure S1**. Sensitivity analyses. (PDF 530 kb)

Additional file 7: **Table S5**. Statistical summary. (PDF 996 kb)

## Abbreviations

CI: Confidence interval
Ctrl: Control group
Ev: Events
Mn: Mandibular interradicular
Mx: Maxillary interradicular
NA: Data not available
NOS: Newcastle-Ottawa scale
OMI: Orthodontic miniscrew implant
pCCT: Prospective controlled clinical trial
pCS: Prospective cohort study
RCT: randomised clinical trial
Trt: Treatment group
VL: Various locations
