# Supplementary material for: Role of anatomical sites and correlated risk factors on the survival of orthodontic miniscrew implants: a systematic review and meta-analysis
Source: Prog Orthod. 2018 Sep 24;19:36. doi: 10.1186/s40510-018-0225-1 (PMC6151309; doi:10.1186/s40510-018-0225-1)
Supplement: Supplementary file 1 — Protocol. (PDF 185 kb) [file 40510_2018_225_MOESM1_ESM.pdf]

Suggested title: Role of anatomical sites and correlated-risk factors on the survival of orthodontic miniscrew implants: a systematic review and meta-analysis

Review question(s)
Where should we insert our orthodontic miniscrew implants?

Searches
A search strategy that uses a combination of controlled vocabulary and free text terms will be developed for each database so as to identify all studies to be considered in this review. An electronic search will be conducted in the following databases:

- Cochrane Central Register of Controlled Trials (CENTRAL)
- Medline
- PubMed
- Scopus
- Web of Knowledge

Other sources will also be searched for ongoing and unpublished studies:

- Dissertation data (www.theses.com)
- Grey literature (www.opengrey.eu)
- ClinicalTrials.gov (http://www.clinicaltrials.gov)
- ISRCTN registry (http://www.controlled-trials.com)

In addition, the reference lists of the included articles will be hand searched for any additional relevant literature altogether with the bibliographies of any other relevant systematic reviews. Conference proceedings and relevant orthodontic journals will be manually searched.
The relevant manufacturers and the corresponding authors of all selected trials will be contacted twice via email to clarify any missing trial details. There will be also no restriction over the publication date in the search.

Types of study to be included
Randomised clinical trials (RCTs) and prospective non-randomised studies are to be included.

Condition or domain being studied
Anatomical locations for orthodontic miniscrew implants.

Participants/ population
Orthodontic patients requiring the insertion of OMIs with no restriction over the type of orthodontic appliance, gender or the presenting age of the patients.

Intervention(s), exposure(s)
Any orthodontic treatment intervention involving the insertion of OMIs at a specific insertion site (interradicular between specific teeth, midpalatal, paramedian, parapalatal, retromolar area or zygomatic buttress).

Comparator(s)/ control
Another insertion site, other forms of treatment or no active treatment.

Outcome(s)

Primary outcomes

Failure rate related to the particular insertion site.

Secondary outcomes

Insertion sites' related risk factors.

Data extraction, (selection and coding)

A flow chart detailing the study identification and selection will be used to present the results. The titles and abstracts of potentially relevant articles will be screened by two reviewers independently and in duplicate. Full text will be examined for eligibility and in case of any discrepancies; a third reviewer will mediate any unresolved disagreement. Data extraction for the included studies will be carried out independently and in duplicate by two reviewers using a pre-piloted data extraction form. Corresponding authors will be contacted if further information will be required. All excluded studies will be listed with their exclusion justification at the exclusion stage.

Risk of bias (quality) assessment

Two independent reviewers will assess the risk of bias within included studies separately. The Cochrane Collaboration's tool will be used to assess the risk of bias within RCTs and the Newcastle-Ottawa scale will be used for the methodological quality judgment of the prospective non-randomised studies. A discussion with a third reviewer will resolve any potential disagreements between the two reviewers.

Strategy for data synthesis

A qualitative synthesis of the findings will be presented in addition to a planned quantitative synthesis of the data whenever similar outcomes are reported in the studies. For dichotomous data, failure rates would be expressed as events and demonstrated as event rates with their corresponding 95% confidence intervals. A random-effects model would be carried out for the pooled data accounting for possible heterogeneity. Heterogeneity would be measured using the I-squared statistic. A 25%, 50% and 75% statistic accounts for low, moderate and high levels of heterogeneity respectively. In addition, publication bias across studies would be assessed visually using the generated funnel plots if more than 10 studies are included.
