# Supplementary material for: Role of anatomical sites and correlated risk factors on the survival of orthodontic miniscrew implants: a systematic review and meta-analysis
Source: Prog Orthod. 2018 Sep 24;19:36. doi: 10.1186/s40510-018-0225-1 (PMC6151309; doi:10.1186/s40510-018-0225-1)
Supplement: Supplementary file 2 — Table S1. Databases, search strategies and exclusions. (PDF 271 kb) [file 40510_2018_225_MOESM2_ESM.pdf]

List of journals and additional bibliographic databases

**Journals searched manually from January 2001 until October 2017**
American Journal of Orthodontics and Dentofacial Orthopedics
Angle Orthodontist
European Journal of Orthodontics
Journal of Orthodontics (and its predecessor, the British Journal of Orthodontics)
Seminars in Orthodontics
Clinical Orthodontics and Research
Australian Orthodontic Journal
Korean Journal of Orthodontics
**Additional bibliographic databases searched until October 2017**
Dissertation data (www.theses.com)
Clinical Trial Registry (www.ClinicalTrials.gov)
ISRCTN registry (https://www.isrctn.com)
Dissertation and Theses Dissemination (http://www.proquest.com)
Grey literature (www.opengrey.eu)
Conference proceedings and abstracts from British, European and Australian Orthodontic
Conferences, International Association for Dental Research – the Conference Papers Index.

List of excluded studies based on their full texts with reasons

Study	Study identification	Reason for exclusion
Cousley 2015	Cousley R. Controlled canine retraction using orthodontic mini-implants coupled with bondable powerarms. Journal of orthodontics. 2015 Oct 2;42(4):315-23.	Case report
Park 2004	Park HS, Bae SM, Kyung HM, Sung JH. Simultaneous incisor retraction and distal molar movement with microimplant anchorage. World journal of orthodontics. 2004 Jun 1;5(2).	Case report
Al-Falahi 2012	Al-Falahi BA, Hammad SM, El-Kenawy MH, Foudad MA. Intrusion of Maxillary Incisors by Mini-screw Anchorage of Angle Class II Division 2 Malocclusion Cases. IJO. 2012;23(4).	Less than 20 inserted miniscrews in a specific site
Basha 2010	Basha AG, Shantaraj R, Mogegowda SB. Comparative study between conventional en-masse retraction (sliding mechanics) and en-masse retraction using orthodontic micro implant. Implant dentistry. 2010 Apr 1;19(2):128-36.	Less than 20 inserted miniscrews in a specific site
Hedayati 2007	Hedayati Z, Hashemi SM, Zamiri B, Fattahi HR. Anchorage value of surgical titanium screws in orthodontic tooth movement. International journal of oral and maxillofacial surgery. 2007 Jul 31;36(7):588-92.	Less than 20 inserted miniscrews in a specific site
Intachai 2009	Intachai I, Krisanaprakornkit S, Kongtawelert P, Ong-chai S, Buranastidporn B, Suzuki EY, Jotikasthira D. Chondroitin sulphate (WF6 epitope) levels in peri-miniscrew implant crevicular fluid during orthodontic loading. The European Journal of Orthodontics. 2009 Jan 1:cjp056.	Less than 20 inserted miniscrews in a specific site
Kircelli 2006	Kircelli BH, Pektas Z, Kircelli C. Maxillary molar distalization with a bone-anchored pendulum appliance. The Angle orthodontist. 2006 Jul;76(4):650-9.	Less than 20 inserted miniscrews in a specific site
Lifshitz 2010	Lifshitz AB, Muñoz M. Evaluation of the stability of self-drilling mini-implants for maxillary anchorage under immediate loading. World journal of orthodontics. 2010 Dec 1;11(4).	Less than 20 inserted miniscrews in a specific site
Luzi 2013	Luzi C, Luzi V, Melsen B. Mini-implants and the efficiency of Herbst treatment: a preliminary study. Progress in orthodontics. 2013 Dec 1;14(1):1-6.	Less than 20 inserted miniscrews in a specific site
Nayak 2011	Nayak UK, Goyal V, Godhrawala F, Saxena R. Comparison of Skeletodental Changes Occurring during Deep Overbite Correction with Mini-Implant Anchorage System and the Utility Arches Reinforced by a Transpalatal Arch. Journal of Indian Orthodontic Society. 2011 Jan 1;45(1):9.	Less than 20 inserted miniscrews in a specific site
Thiruvenkatachari 2006	Thiruvenkatachari B, Pavithranand A, Rajasigamani K, Kyung HM. Comparison and measurement of the amount of anchorage loss of the molars with and without the use of implant anchorage during canine retraction. American journal	Less than 20 inserted miniscrews in a specific site

	of orthodontics and dentofacial orthopedics. 2006 Apr 30;129(4):551-4.	
Wahabuddin 2015	Wahabuddin S, Mascarenhas R, Iqbal M, Husain A. Clinical application of micro-implant anchorage in initial orthodontic retraction. Journal of Oral Implantology. 2015 Feb;41(1):77-84.	Less than 20 inserted miniscrews in a specific site
Wilmes 2009	Wilmes B, Olthoff G, Drescher D. Comparison of skeletal and conventional anchorage methods in conjunction with pre-operative decompensation of a skeletal class III malocclusion. Journal of Orofacial Orthopedics/Fortschritte der Kieferorthopädie. 2009 Jul 1;70(4):297-305.	Less than 20 inserted miniscrews in a specific site
Abbassy 2015	Abbassy MA, Sabban HM, Hassan AH, Zawawi KH. Evaluation of mini-implant sites in the posterior maxilla using traditional radiographs and cone-beam computed tomography. Saudi medical journal. 2015 Nov;36(11):1336.	No insertion site specific failure rate
Arantes 2012	Arantes FD, Kina J, Gonçalves MJ, Gurgel JD, Silva Filho OG, Santos EC. Mini-implant and Nance button for initial retraction of maxillary canines: a prospective study in cast models. Dental Press Journal of Orthodontics. 2012 Aug;17(4):134-9.	No insertion site specific failure rate
Apel 2009	Apel S, Apel C, Morea C, Tortamano A, Dominguez GC, Conrads G. Microflora associated with successful and failed orthodontic mini-implants. Clinical oral implants research. 2009 Nov 1;20(11):1186-90.	No insertion site specific failure rate
Bayat 2010	Bayat E. Effect of smoking on the failure rates of orthodontic miniscrews. Journal of Orofacial Orthopedics/Fortschritte der Kieferorthopädie. 2010 Mar 1;71(2):117-24.	No insertion site specific failure rate
Bratu 2014	Bratu DC, Popa G, Petrescu H, Karancsi OL, Bratu EA. Influence of Chemically-Modified Implant Surfaces on the Stability of Orthodontic Mini-Implants. REVISTA DE CHIMIE. 2014 Oct 1;65(10):1222-5.	No insertion site specific failure rate
Bahl-Palomo 2007	Bahl-Palomo L, Bissada N, Palomo JM, Hans MG. Image guided placement of temporary anchorage devices for tooth movement. International Journal of Computer Assisted Radiology and Surgery (Print). 2007;2(Suppl. 1):S424-6.	No insertion site specific failure rate
Baxmann 2010	Baxmann M, McDonald F, Bourauel C, Jäger A. Expectations, acceptance, and preferences regarding microimplant treatment in orthodontic patients: A randomized controlled trial. American Journal of Orthodontics and Dentofacial Orthopedics. 2010 Sep 30;138(3):250-e1.	No insertion site specific failure rate
Berens 2005	Berens A, Wiechmann D, Rüdiger J. L'ancrage intra-osseux en orthodontie à l'aide de mini-et de microvis. International orthodontics. 2005 Sep 30;3(3):235-43.	No insertion site specific failure rate
Berens 2006	Berens A, Wiechmann D, Dempf R. Mini-and micro-screws for temporary skeletal anchorage in orthodontic therapy. Journal of Orofacial Orthopedics/Fortschritte der	No insertion site specific failure rate

	Kieferorthopädie. 2006 Nov 1;67(6):450-8.	
Bearn 2015	Bearn DR, Alharbi F. British Orthodontic Society national audit of temporary anchorage devices (TADs): report of the first thousand TADs placed. Journal of orthodontics. 2015 Sep 1;42(3):214-9.	No insertion site specific failure rate
Bhalla 2013	Bhalla K, Kalha AS. Miniscrew design and bone response: defining a correlation. ORTHODONTICS: The Art & Practice of Dentofacial Enhancement. 2013 Mar 1;14(1).	No insertion site specific failure rate
Calderon 2011	Calderón JH, Valencia RM, Casasa AA, Sánchez MA, Espinosa R, Ceja I. Biomechanical anchorage evaluation of mini-implants treated with sandblasting and acid etching in orthodontics. Implant dentistry. 2011 Aug 1;20(4):273-9.	No insertion site specific failure rate
Chaddad 2008	Chaddad K, Ferreira AH, Geurs N, Reddy MS. Influence of surface characteristics on survival rates of mini-implants. The Angle orthodontist. 2008 Jan;78(1):107-13.	No insertion site specific failure rate
Chen 2015	Chen M, Li ZM, Liu X, Cai B, Wang DW, Feng ZC. Differences of treatment outcomes between self-ligating brackets with microimplant and headgear anchorages in adults with bimaxillary protrusion. American Journal of Orthodontics and Dentofacial Orthopedics. 2015 Apr 30;147(4):465-71.	No insertion site specific failure rate
Chen 2012	Chen Y, Hong L, Wang CL, Zhang SJ, Cao C, Wei F, Lv T, Zhang F, Liu DX. Effect of large incisor retraction on upper airway morphology in adult bimaxillary protrusion patients: Three-dimensional multislice computed tomography registration evaluation. The Angle orthodontist. 2012 Mar 30;82(6):964-70.	No insertion site specific failure rate
Chopra 2015	Chopra SS, Chakranarayan A. Clinical evaluation of immediate loading of titanium orthodontic implants. medical journal armed forces india. 2015 Apr 30;71(2):165-70.	No insertion site specific failure rate
Cozzani 2010	Cozzani M, Zallio F, Lombardo L, Gracco A. Efficiency of the distal screw in the distal movement of maxillary molars. World J Orthod. 2010 Dec 1;11:341-5.	No insertion site specific failure rate
Deguchi 2008	Deguchi T, Murakami T, Kuroda S, Yabuuchi T, Kamioka H, Takano-Yamamoto T. Comparison of the intrusion effects on the maxillary incisors between implant anchorage and J-hook headgear. American Journal of Orthodontics and Dentofacial Orthopedics. 2008 May 31;133(5):654-60.	No insertion site specific failure rate
Du 2015	Du JK, Tseng YC, Chen HS, Chen CM, Wu JH. Patient's Perception of Pain in Treatment with Temporary Anchorage Devices (Micro-implant, Mini-implant and Mini-plate): Intragroup and Intergroup Analysis. Age. 2015 Apr 16;2(15/3).	No insertion site specific failure rate
Dobranszki 2014	Dobranszki A, Faber J, Scatolino IV, Dobranszki NP, Cordeiro A, Toledo OA. Analysis of factors associated with orthodontic microscrew failure. Brazilian dental journal.	No insertion site specific failure rate

	2014;25(4):346-51.	
Elkordy 2015	Elkordy SA, Fayed MM, Abouelezz AM, Attia KH. Comparison of patient acceptance of the Forsus Fatigue Resistant Device with and without mini-implant anchorage: A randomized controlled trial. American Journal of Orthodontics and Dentofacial Orthopedics. 2015 Nov 30;148(5):755-64.	No insertion site specific failure rate
Ersahan 2015	Ersahan S, Sabuncuoglu FA. Effects of magnitude of intrusive force on pulpal blood flow in maxillary molars. American Journal of Orthodontics and Dentofacial Orthopedics. 2015 Jul 31;148(1):83-9.	No insertion site specific failure rate
Estelita 2009	Estelita S, Janson G, Chiqueto K, Janson M, de Freitas MR. Predictable drill-free screw positioning with a graduated 3-dimensional radiographic-surgical guide: a preliminary report. American Journal of Orthodontics and Dentofacial Orthopedics. 2009 Nov 30;136(5):722-35.	No insertion site specific failure rate
Falkensammer 2014	Falkensammer F, Rausch-Fan X, Arnhart C, Krall C, Schaden W, Freudenthaler J. Impact of extracorporeal shock-wave therapy on the stability of temporary anchorage devices in adults: A single-center, randomized, placebo-controlled clinical trial. American Journal of Orthodontics and Dentofacial Orthopedics. 2014 Oct 31;146(4):413-22.	No insertion site specific failure rate
Falkensammer 2014	Falkensammer F, Arnhart C, Krall C, Schaden W, Freudenthaler J, Bantleon HP. Impact of extracorporeal shock wave therapy (ESWT) on orthodontic tooth movement—a randomized clinical trial. Clinical oral investigations. 2014 Dec 1;18(9):2187-92.	No insertion site specific failure rate
Feldmann 2007	Feldmann I, List T, Feldmann H, Bondemark L. Pain intensity and discomfort following surgical placement of orthodontic anchoring units and premolar extraction: a randomized controlled trial. The Angle orthodontist. 2007 Jul;77(4):578-85.	No insertion site specific failure rate
Feldmann 2012	Feldmann I, List T, Bondemark L. Orthodontic anchoring techniques and its influence on pain, discomfort, and jaw function—a randomized controlled trial. The European Journal of Orthodontics. 2012 Feb 1;34(1):102-8.	No insertion site specific failure rate
Foot 2014	Foot R, Dalci O, Gonzales C, Tarraf NE, Darendeliler MA. The short-term skeleto-dental effects of a new spring for the intrusion of maxillary posterior teeth in open bite patients. Progress in orthodontics. 2014 Sep 25;15(1):1.	No insertion site specific failure rate
Freudenthaler 2001	Freudenthaler JW, Bantleon HP, Haas R. Bicortical titanium screws for critical orthodontic anchorage in the mandible: a preliminary report on clinical applications. Clinical oral implants research. 2001 Aug 1;12(4):358-63.	No insertion site specific failure rate
Fritz 2004	Fritz PD, Ehmer A, Diedrich P. Clinical suitability of titanium microscrews for orthodontic anchorage—preliminary	No insertion site specific failure rate

	experiences. Journal of Orofacial Orthopedics/Fortschritte der Kieferorthopädie. 2004 Sep 1;65(5):410-8.	
Garg 2015	Garg KK, Gupta M. Assessment of stability of orthodontic mini-implants under orthodontic loading: A computed tomography study. Indian Journal of Dental Research. 2015 May 1;26(3):237.	No insertion site specific failure rate
Hamamci 2011	Hamamcı N, Kaya FA, Uysal E, Yokuş B. Identification of interleukin 2, 6, and 8 levels around miniscrews during orthodontic tooth movement. The European Journal of Orthodontics. 2011 Apr 7:cjr019.	No insertion site specific failure rate
Heravi 2011	Heravi F, Bayani S, Madani AS, Radvar M, Anbiaee N. Intrusion of supra-erupted molars using miniscrews: clinical success and root resorption. American Journal of Orthodontics and Dentofacial Orthopedics. 2011 Apr 30;139(4):S170-5.	No insertion site specific failure rate
Hourfar 2017	Hourfar J, Bister D, Lux CJ, Al-Tamimi B, Ludwig B. Anatomic landmarks and availability of bone for placement of orthodontic mini-implants for normal and short maxillary body lengths. American Journal of Orthodontics and Dentofacial Orthopedics. 2017 May 31;151(5):878-86.	No insertion site specific failure rate
Inoue 2014	Inoue M, Kuroda S, Yasue A, Horiuchi S, Kyung HM, Tanaka E. Torque ratio as a predictable factor on primary stability of orthodontic miniscrew implants. Implant dentistry. 2014 Oct 1;23(5):576-81.	No insertion site specific failure rate
Jain 2014	Jain RK, Kumar SP, Manjula WS. Comparison of intrusion effects on maxillary incisors among mini implant anchorage, j-hook headgear and utility arch. J Clin Diagn Res. 2014 Jul;8(7):21-4.	No insertion site specific failure rate
Jang 2009	Jang I, Tanaka M, Koga Y, Iijima S, Yozgatian JH, Cha BK, Yoshida N. A novel method for the assessment of three-dimensional tooth movement during orthodontic treatment. The Angle Orthodontist. 2009 May;79(3):447-53.	No insertion site specific failure rate
Jeong 2015	Jeong JW, Kim JW, Lee NK, Kim YK, Lee JH, Kim TW. Analysis of time to failure of orthodontic mini-implants after insertion or loading. Journal of the Korean Association of Oral and Maxillofacial Surgeons. 2015 Oct 1;41(5):240-5.	No insertion site failure rate
Jung 2012	Jung MH. A comparison of second premolar extraction and mini-implant total arch distalization with interproximal stripping. The Angle Orthodontist. 2012 Nov 30;83(4):680-5.	No insertion site specific failure rate
Jung 2013	Jung YR, Kim SC, Kang KH, Cho JH, Lee EH, Chang NY, Chae JM. Placement angle effects on the success rate of orthodontic microimplants and other factors with cone-beam computed tomography. American Journal of Orthodontics and Dentofacial Orthopedics. 2013 Feb 28;143(2):173-81.	No insertion site specific failure rate
Justens 2008	Justens E, De Bruyn H. Clinical Outcome of Mini-Screws Used as Orthodontic Anchorage. Clinical implant dentistry	No insertion site specific failure rate

	and related research. 2008 Sep 1;10(3):174-80.	
Kaya 2011	Kaya FA, Hamamcı N, Uysal E, Yokuş B. Identification of tumor necrosis factor-α levels around miniscrews during canine distalization. Korean Journal of Orthodontics. 2011 Feb 1;41(1):36-41.	No insertion site specific failure rate
Khanna 2014	Khanna R, Tikku T, Sachan K, Maurya RP, Verma G, Ojha V. Evaluation of canine retraction following periodontal distraction using NiTi coil spring and implants–A clinical study. Journal of Oral Biology and Craniofacial Research. 2014 Dec 31;4(3):192-9.	No insertion site specific failure rate
Kinzinger 2008	Kinzinger PD, Gülden N, Yildizhan F, Hermanns-Sachweh B, Diedrich P. Anchorage efficacy of palatally-inserted miniscrews in molar distalization with a periodontally/miniscrew-anchored distal jet. Journal of Orofacial Orthopedics/Fortschritte der Kieferorthopädie. 2008 Mar 1;69(2):110-20.	No insertion site specific failure rate
Kinzinger 2009	Kinzinger GS, Gülden N, Yildizhan F, Diedrich PR. Efficiency of a skeletonized distal jet appliance supported by miniscrew anchorage for noncompliance maxillary molar distalization. American Journal of Orthodontics and Dentofacial Orthopedics. 2009 Oct 31;136(4):578-86.	No insertion site specific failure rate
Kuroda 2007	Kuroda S, Sakai Y, Tamamura N, Deguchi T, Takano-Yamamoto T. Treatment of severe anterior open bite with skeletal anchorage in adults: comparison with orthognathic surgery outcomes. American Journal of Orthodontics and Dentofacial Orthopedics. 2007 Nov 30;132(5):599-605.	No insertion site specific failure rate
Kuroda 2009	Kuroda S, Yamada K, Deguchi T, Kyung HM, Takano-Yamamoto T. Class II malocclusion treated with miniscrew anchorage: comparison with traditional orthodontic mechanics outcomes. American Journal of Orthodontics and Dentofacial Orthopedics. 2009 Mar 31;135(3):302-9.	No insertion site specific failure rate
Kokitsawat 2008	Kokitsawat S, Manosudprasit M, Godfrey K, Chatchaiwiwattana C. Clinical effects associated with miniscrews used as orthodontic anchorage. Australian orthodontic journal. 2008 Nov;24(2):134.	No insertion site specific failure rate
Kocsis 2012	Kocsis A, Seres L. Orthodontic screws to extrude impacted maxillary canines. Journal of Orofacial Orthopedics/Fortschritte der Kieferorthopädie. 2012 Jan 1;73(1):19-27.	No insertion site specific failure rate
Lagravère 2010	Lagravère MO, Carey J, Heo G, Toogood RW, Major PW. Transverse, vertical, and anteroposterior changes from bone-anchored maxillary expansion vs traditional rapid maxillary expansion: a randomized clinical trial. American Journal of Orthodontics and Dentofacial Orthopedics. 2010 Mar 31;137(3):304-e1.	No insertion site specific failure rate
Lagravere 2013	Lagravère MO, Gamble J, Major PW, Heo G. Transverse	No insertion site specific

	dental changes after tooth-borne and bone-borne maxillary expansion. International Orthodontics. 2013 Mar 31;11(1):21-34.	failure rate
Lehnen 2011	Lehnen S, McDonald F, Bourauel C, Baxmann M. Patient expectations, acceptance and preferences in treatment with orthodontic mini-implants. Journal of Orofacial Orthopedics/Fortschritte der Kieferorthopädie. 2011 Apr 1;72(2):93-102.	No insertion site specific failure rate
Lee 2016	Lee MY, Park JH, Kim SC, Kang KH, Cho JH, Cho JW, Chang NY, Chae JM. Bone density effects on the success rate of orthodontic microimplants evaluated with cone-beam computed tomography. American Journal of Orthodontics and Dentofacial Orthopedics. 2016 Feb 29;149(2):217-24.	No insertion site specific failure rate
Lee 2012	Lee SJ, Jang SY, Chun YS, Lim WH. Three-dimensional analysis of tooth movement after intrusion of a supraerupted molar using a mini-implant with partial-fixed orthodontic appliances. The Angle Orthodontist. 2012 Sep 4;83(2):274-9.	No insertion site specific failure rate
Mah 2016	Mah SJ, Kim JE, Ahn EJ, Nam JH, Kim JY, Kang YG. Analysis of midpalatal miniscrew-assisted maxillary molar distalization patterns with simultaneous use of fixed appliances: A preliminary study. The Korean Journal of Orthodontics. 2016 Jan 1;46(1):55-61.	No insertion site specific failure rate
Ma 2008	Ma J, Wang L, Zhang W, Chen W, Zhao C, Smales RJ. Comparative evaluation of micro-implant and headgear anchorage used with a pre-adjusted appliance system. European journal of orthodontics. 2008 Jun 1;30(3):283.	No insertion site specific failure rate
Maino 2012	Maino BG, Pagin P, Di Blasio A. Success of miniscrews used as anchorage for orthodontic treatment: analysis of different factors. Progress in orthodontics. 2012 Nov 30;13(3):202-9.	No insertion site specific failure rate
Melo 2012	Melo AC, Duarte DS, Shimizu RH, Campos D, Andrighetto AR. Lower molar uprighting with miniscrew anchorage: direct and indirect anchorage. International journal of orthodontics (Milwaukee, Wis.). 2012 Dec;24(3):9-14.	No insertion site specific failure rate
Min 2012	Min KI, Kim SC, Kang KH, Cho JH, Lee EH, Chang NY, Chae JM. Root proximity and cortical bone thickness effects on the success rate of orthodontic micro-implants using cone beam computed tomography. The Angle orthodontist. 2012 Mar 14;82(6):1014-21.	No insertion site specific failure rate
Migliorati 2016	Migliorati M, Drago S, Gallo F, Amorfini L, Dalessandri D, Benedicenti S, Silvestrini-Biavati A. Immediate versus delayed loading: comparison of primary stability loss after miniscrew placement in orthodontic patients—a single-centre blinded randomized clinical trial. The European Journal of Orthodontics. 2016 Jan 4:cjv095.	No insertion site specific failure rate
Motoyoshi 2006	Motoyoshi M, Hirabayashi M, Uemura M, Shimizu N. Recommended placement torque when tightening an	No insertion site specific failure rate

	orthodontic mini-implant. Clinical oral implants research. 2006 Feb 1;17(1):109-14.	
Motoyoshi 2007	Motoyoshi M, Yoshida T, Ono A, Shimizu N. Effect of cortical bone thickness and implant placement torque on stability of orthodontic mini-implants. International Journal of Oral & Maxillofacial Implants. 2007 Sep 1;22(5).	No insertion site specific failure rate
Motoyoshi 2007	Motoyoshi M, Matsuoka M, Shimizu N. Application of orthodontic mini-implants in adolescents. International journal of oral and maxillofacial surgery. 2007 Aug 31;36(8):695-9.	No insertion site specific failure rate
Motoyoshi 2010	Motoyoshi M, Uemura M, Ono A, Okazaki K, Shigeeda T, Shimizu N. Factors affecting the long-term stability of orthodontic mini-implants. American Journal of Orthodontics and Dentofacial Orthopedics. 2010 May 31;137(5):588-e1.	No insertion site specific failure rate
Ngiam 2012	Ngiam J, Kyung HM. Microimplant-based mandibular advancement therapy for the treatment of snoring and obstructive sleep apnea: a prospective study. The Angle Orthodontist. 2012 May 10;82(6):978-84.	No insertion site specific failure rate
Nienkemper 2013	Nienkemper M, Wilmes B, Pauls A, Drescher D. Impact of mini-implant length on stability at the initial healing period: a controlled clinical study. Head & face medicine. 2013 Oct 20;9(1):1.	No insertion site specific failure rate
Nienkemper 2013	Nienkemper M, Wilmes B, Pauls A, Drescher D. Mini-implant stability at the initial healing period: a clinical pilot study. The Angle Orthodontist. 2013 Jul 24;84(1):127-33.	No insertion site specific failure rate
Nienkemper 2015	Nienkemper M, Pauls A, Ludwig B, Drescher D. Stability of paramedian inserted palatal mini-implants at the initial healing period: a controlled clinical study. Clinical oral implants research. 2015 Aug 1;26(8):870-5.	No insertion site specific failure rate
Oh 2011	Oh YH, Park HS, Kwon TG. Treatment effects of microimplant-aided sliding mechanics on distal retraction of posterior teeth. American Journal of Orthodontics and Dentofacial Orthopedics. 2011 Apr 30;139(4):470-81.	No insertion site specific failure rate
Ono 2008	Ono A, Motoyoshi M, Shimizu N. Cortical bone thickness in the buccal posterior region for orthodontic mini-implants. International journal of oral and maxillofacial surgery. 2008 Apr 30;37(4):334-40.	No insertion site specific failure rate
Ozdemir 2015	Ozdemir F, Demir HB, Oztoprak MO, Tozlu M. A report on the use of Er: YAG laser for pilot hole drilling prior to miniscrew insertion. Lasers in medical science. 2015 Feb 1;30(2):605-9.	No insertion site specific failure rate
Pavlin 2015	Pavlin D, Anthony R, Raj V, Gakunga PT. Cyclic loading (vibration) accelerates tooth movement in orthodontic patients: a double-blind, randomized controlled trial. InSeminars in Orthodontics 2015 Sep 30 (Vol. 21, No. 3, pp. 187-194). WB Saunders.	No insertion site specific failure rate

Pithon 2015	Pithon MM, Santos MJ, Ribeiro MC, Nascimento RC, Rodrigues RS, Ruellas AC, Coqueiro RS. Patients' perception of installation, use and results of orthodontic mini-implants. Acta odontologica latinoamericana: AOL. 2015 Aug;28(2):108-12.	No insertion site specific failure rate
Poon 2015	Poon YC, Chang HP, Tseng YC, Chou ST, Cheng JH, Liu PH, Pan CY. Palatal bone thickness and associated factors in adult miniscrew placements: A cone-beam computed tomography study. The Kaohsiung journal of medical sciences. 2015 May 31;31(5):265-70.	No insertion site specific failure rate
Polat-Özsoy 2011	Polat-Özsoy Ö, Arman-Özçırpıcı A, Veziroğlu F, Çetinşahin A. Comparison of the intrusive effects of miniscrews and utility arches. American Journal of Orthodontics and Dentofacial Orthopedics. 2011 Apr 30;139(4):526-32.	No insertion site specific failure rate
Raj 2015	Raj A, Acharya SS, Mohanty P, Prabhakar R, Karthikeyan MK, Saravanan R, Vikram NR. Comparison of Intrusive Effects of Mini screws and Burrstone Intrusive Arch: A Radiographic Study. Advances In Human Biology. 2015 Aug 30;5(2):49-55.	No insertion site specific failure rate
Reznik 2009	Reznik DS, Jeske AH, Chen JW, English J. Comparative efficacy of 2 topical anesthetics for the placement of orthodontic temporary anchorage devices. Anesthesia progress. 2009 Sep;56(3):81-5.	No insertion site specific failure rate
Rudzki-Janson 2008	Rudzki-Janson I, Stockmann P, Schlegel KA, Végh A. Immediate loading of palatal implants in stillgrowing patients: A prospective, comparative, clinical pilot study. Journal of Orofacial Orthopedics/Fortschritte der Kieferorthopädie. 2008 Jul 1;69(4):297-308.	No insertion site specific failure rate
Sabuncuoglu 2014	Sabuncuoglu FA, Ersahan S. Changes in maxillary molar pulp blood flow during orthodontic intrusion. Australian orthodontic journal. 2014 Nov 1;30(2):152-60.	No insertion site specific failure rate
Sar 2012	Sar C, Kaya B, Ozsoy O, Özcirpici AA. Comparison of two implant-supported molar distalization systems. The Angle Orthodontist. 2012 Oct 29;83(3):460-7.	No insertion site specific failure rate
Sandler 2014	Sandler J, Murray A, Thiruvenkatachari B, Gutierrez R, Speight P, O'Brien K. Effectiveness of 3 methods of anchorage reinforcement for maximum anchorage in adolescents: A 3-arm multicenter randomized clinical trial. American Journal of Orthodontics and Dentofacial Orthopedics. 2014 Jul 31;146(1):10-20.	No insertion site specific failure rate
Santiago 2009	Santiago RC, de Paula FO, Fraga MR, Assis NM, Vitral RW. Correlation between miniscrew stability and bone mineral density in orthodontic patients. American Journal of Orthodontics and Dentofacial Orthopedics. 2009 Aug 31;136(2):243-50.	No insertion site specific failure rate
Schätzle 2009	Schätzle M, Männchen R, Balbach U, Hämmerle CH,	No insertion site specific

	Toutenburg H, Jung RE. Stability change of chemically modified sandblasted/acid-etched titanium palatal implants. A randomized-controlled clinical trial. Clinical oral implants research. 2009 May 1;20(5):489-95.	failure rate
Park 2008	Park HS, Yoon DY, Park CS, Jeoung SH. Treatment effects and anchorage potential of sliding mechanics with titanium screws compared with the Tweed-Merrifield technique. American Journal of Orthodontics and Dentofacial Orthopedics. 2008 Apr 30;133(4):593-600.	No insertion site specific failure rate
Tausche 2007	Tausche E, Hansen L, Hietschold V, Lagravère MO, Harzer W. Three-dimensional evaluation of surgically assisted implant bone-borne rapid maxillary expansion: a pilot study. American Journal of Orthodontics and Dentofacial Orthopedics. 2007 Apr 30;131(4):S92-9.	No insertion site specific failure rate
Tasanapanont 2017	Tasanapanont J, Wattanachai T, Apisariyakul J, Pothacharoen P, Ongchai S, Kongtawelert P, Midtbø M, Jotikasthira D. Biochemical and Clinical Assessments of Segmental Maxillary Posterior Tooth Intrusion. International journal of dentistry. 2017 Feb 22;2017.	No insertion site specific failure rate
Thiruvenkatachari 2008	Thiruvenkatachari B, Ammayappan P, Kandaswamy R. Comparison of rate of canine retraction with conventional molar anchorage and titanium implant anchorage. American Journal of Orthodontics and Dentofacial Orthopedics. 2008 Jul 31;134(1):30-5.	No insertion site specific failure rate
Upadhyay 2008	Upadhyay M, Yadav S, Nagaraj K, Patil S. Treatment effects of mini-implants for en-masse retraction of anterior teeth in bialveolar dental protrusion patients: a randomized controlled trial. American Journal of Orthodontics and Dentofacial Orthopedics. 2008 Jul 31;134(1):18-29.	No insertion site specific failure rate
Upadhay 2011	Upadhyay M, Yadav S, Nagaraj K, Uribe F, Nanda R. Mini-implants vs fixed functional appliances for treatment of young adult Class II female patients: a prospective clinical trial. The Angle orthodontist. 2011 Aug 26;82(2):294-303.	No insertion site specific failure rate
Vasoglou 2014	Vasoglou M, Chrysomali E, Zinelis S, Bitsanis I, Haralambakis N, Makou M, Eliades G. Retrieval analysis of immediately loaded orthodontic mini-implants: material and tissue characterization. The European Journal of Orthodontics. 2014 Dec 1;36(6):683-9.	No insertion site specific failure rate
Victor 2014	Victor D, Prabhakar R, Karthikeyan MK, Saravanan R, Vanathi P, Vikram NR, Reddy PA, Sudeepthi M. Effectiveness of Mini Implants in Three-Dimensional Control During Retraction-A Clinical Study. Journal of clinical and diagnostic research: JCDR. 2014 Feb;8(2):227.	No insertion site specific failure rate
Virang 2013	Virang B, Makhija PG, Belludi A, Bhatia V, Padmawar SS, Gupta A. Evaluation of Titanium Miniscrew Implants as a Source of Intraoral Anchorage for en masse Intrusion of	No insertion site specific failure rate

	Maxillary Anterior Teeth: A Clinical Study. The Journal of Indian Orthodontic Society. 2013 Oct 1;47(4):184.	
Wang 2012	Wang Q, Chen W, Smales RJ, Peng H, Hu X, Yin L. Apical root resorption in maxillary incisors when employing micro-implant and J-hook headgear anchorage: a 4-month radiographic study. Journal of Huazhong University of Science and Technology. Medical sciences= Hua zhong ke ji da xue xue bao. Yi xue Ying De wen ban= Huazhong keji daxue xuebao. Yixue Yingdewen ban. 2012 Oct;32(5):767.	No insertion site specific failure rate
Wiechmann 2007	Wiechmann D, Meyer U, Büchter A. Success rate of mini-and micro-implants used for orthodontic anchorage: a prospective clinical study. Clinical oral implants research. 2007 Apr 1;18(2):263-7.	No insertion site specific failure rate
Xun 2007	Xun C, Zeng X, Wang X. Microscrew anchorage in skeletal anterior open-bite treatment. The Angle orthodontist. 2007 Jan;77(1):47-56.	No insertion site specific failure rate
Yang 2015	Yang L, Li F, Cao M, Chen H, Wang X, Chen X, Yang L, Gao W, Petrone JF, Ding Y. Quantitative evaluation of maxillary interradicular bone with cone-beam computed tomography for bicortical placement of orthodontic mini-implants. American Journal of Orthodontics and Dentofacial Orthopedics. 2015 Jun 30;147(6):725-37.	No insertion site specific failure rate
Yamada 2009	Yamada K, Kuroda S, Deguchi T, Takano-Yamamoto T, Yamashiro T. Distal movement of maxillary molars using miniscrew anchorage in the buccal interradicular region. The Angle orthodontist. 2009 Jan;79(1):78-84.	No insertion site specific failure rate
Ye 2013	Ye C, Zhihe Z, Zhao Q, Ye J. Treatment effects of distal movement of lower arch with miniscrews in the retromolar area compared with miniscrews in the posterior area of the maxillary. Journal of Craniofacial Surgery. 2013 Nov 1;24(6):1974-9.	No insertion site specific failure rate
Yoo 2014	Yoo SH, Park YC, Hwang CJ, Kim JY, Choi EH, Cha JY. A comparison of tapered and cylindrical miniscrew stability. The European Journal of Orthodontics. 2014 Oct 1;36(5):557-62.	No insertion site specific failure rate
Arcuri 2007	Arcuri C, Muzzi F, Santini F, Barlattani A, Giancotti A. Five years of experience using palatal mini-implants for orthodontic anchorage. Journal of Oral and Maxillofacial Surgery. 2007 Dec 31;65(12):2492-7.	Retrospective design
Baik 2015	Baik UB, Kook YA, Bayome M, Park JU, Park JH. Vertical eruption patterns of impacted mandibular third molars after the mesialization of second molars using miniscrews. The Angle Orthodontist. 2015 Oct 6.	Retrospective design
Baek 2008	Baek SH, Kim BM, Kyung SH, Lim JK, Kim YH. Success rate and risk factors associated with mini-implants reinstalled in the maxilla. The Angle Orthodontist. 2008 Sep;78(5):895-	Retrospective design

	901.	
Carano 2004	Carano A, Velo S, Incorvati C, Poggio P. Clinical applications of the Mini-Screw-Anchorage-System (MAS) in the maxillary alveolar bone. Prog Orthod. 2004;5(2):212-35.	Retrospective design
Chang 2015	Chang C, Liu SS, Roberts WE. Primary failure rate for 1680 extra-alveolar mandibular buccal shelf mini-screws placed in movable mucosa or attached gingiva. The Angle Orthodontist. 2015 Jan 20;85(6):905-10.	Retrospective design
Chen 2006	Chen CH, Chang CS, Hsieh CH, Tseng YC, Shen YS, Huang IY, Yang CF, Chen CM. The use of microimplants in orthodontic anchorage. Journal of Oral and Maxillofacial Surgery. 2006 Aug 31;64(8):1209-13.	Retrospective design
Cozzani 2016	Cozzani M, Fontana M, Maino G, Maino G, Palpacelli L, Caprioglio A. Comparison between direct vs indirect anchorage in two miniscrew-supported distalizing devices. The Angle Orthodontist. 2016 May;86(3):399-406.	Retrospective design
Da Costa 2015	da Costa Sabec R, Fernandes TM, de Lima Navarro R, Oltramari-Navarro PV, Conti AC, de Almeida MR, Poleti ML. Can bone thickness and inter-radicular space affect miniscrew placement in posterior mandibular sites?. Journal of Oral and Maxillofacial Surgery. 2015 Feb 28;73(2):333-9.	Retrospective design
Deguchi 2011	Deguchi T, Kurosaka H, Oikawa H, Kuroda S, Takahashi I, Yamashiro T, Takano-Yamamoto T. Comparison of orthodontic treatment outcomes in adults with skeletal open bite between conventional edgewise treatment and implant-anchored orthodontics. American Journal of Orthodontics and Dentofacial Orthopedics. 2011 Apr 30;139(4):S60-8.	Retrospective design
He 2013	He S, Gao J, Wamalwa P, Wang Y, Zou S, Chen S. Camouflage treatment of skeletal Class III malocclusion with multiloop edgewise arch wire and modified Class III elastics by maxillary mini-implant anchorage. The Angle Orthodontist. 2013 Jan 11;83(4):630-40.	Retrospective design
Hourfar 2015	Hourfar J, Ludwig B, Bister D, Braun A, Kanavakis G. The most distal palatal ruga for placement of orthodontic mini-implants. The European Journal of Orthodontics. 2015 Aug 1;37(4):373-8.	Retrospective design
Jamilian 2011	Jamilian A, Haraji A, Showkatbakhsh R, Valaee N. The effects of miniscrew with Class III traction in growing patients with maxillary deficiency. IJO. 2011;22(1).	Retrospective design
Jee 2014	Jee JH, Ahn HW, Seo KW, Kim SH, Kook YA, Chung KR, Nelson G. En-masse retraction with a preformed nickel-titanium and stainless steel archwire assembly and temporary skeletal anchorage devices without posterior bonding. The Korean Journal of Orthodontics. 2014 Sep 1;44(5):236-45.	Retrospective design
Kuroda 2007	Kuroda S, Yamada K, Deguchi T, Hashimoto T, Kyung HM, Yamamoto TT. Root proximity is a major factor for screw	Retrospective design

	failure in orthodontic anchorage. American Journal of Orthodontics and Dentofacial Orthopedics. 2007 Apr 30;131(4):S68-73.	
Koyama 2010	Koyama I, Iino S, Abe Y, Takano-Yamamoto T, Miyawaki S. Differences between sliding mechanics with implant anchorage and straight-pull headgear and intermaxillary elastics in adults with bimaxillary protrusion. The European Journal of Orthodontics. 2010 Nov 8;33(2):126-31.	Retrospective design
Kim 2010	Kim YH, Yang SM, Kim S, Lee JY, Kim KE, Gianelly AA, Kyung SH. Midpalatal miniscrews for orthodontic anchorage: factors affecting clinical success. American Journal of Orthodontics and Dentofacial Orthopedics. 2010 Jan 31;137(1):66-72.	Retrospective design
Lim 2009	Lim HJ, Eun CS, Cho JH, Lee KH, Hwang HS. Factors associated with initial stability of miniscrews for orthodontic treatment. American Journal of Orthodontics and Dentofacial Orthopedics. 2009 Aug 31;136(2):236-42.	Retrospective design
Lee 2010	Lee SJ, Ahn SJ, Lee JW, Kim SH, Kim TW. Survival analysis of orthodontic mini-implants. American Journal of Orthodontics and Dentofacial Orthopedics. 2010 Feb 28;137(2):194-9.	Retrospective design
Lee 2013	Lee J, Miyazawa K, Tabuchi M, Kawaguchi M, Shibata M, Goto S. Midpalatal miniscrews and high-pull headgear for anteroposterior and vertical anchorage control: cephalometric comparisons of treatment changes. American Journal of Orthodontics and Dentofacial Orthopedics. 2013 Aug 31;144(2):238-50.	Retrospective design
Lee 2011	Lee AY, Kim YH. Comparison of movement of the upper dentition according to anchorage method: orthodontic mini-implant versus conventional anchorage reinforcement in class I malocclusion. ISRN dentistry. 2010 Dec 23;2011.	Retrospective design
Lin 2014	Lin L, Ahn HW, Kim SJ, Moon SC, Kim SH, Nelson G. Tooth-borne vs bone-borne rapid maxillary expanders in late adolescence. The Angle Orthodontist. 2014 Jun 19;85(2):253-62.	Retrospective design
Manni 2014	Manni A, Pasini M, Mazzotta L, Mutinelli S, Nuzzo C, Grassi FR, Cozzani M. Comparison between an acrylic splint herbst and an acrylic splint miniscrew-herbst for mandibular incisors proclination control. International journal of dentistry. 2014 May 19;2014.	Retrospective design
Mariani 2014	Mariani L, Maino G, Caprioglio A. Skeletal versus conventional intraoral anchorage for the treatment of class II malocclusion: dentoalveolar and skeletal effects. Progress in orthodontics. 2014 Jul 30;15(1):1.	Retrospective design
Moon 2008	Moon CH, Lee DG, Lee HS, Im JS, Baek SH. Factors associated with the success rate of orthodontic miniscrews	Retrospective design

	placed in the upper and lower posterior buccal region. The Angle orthodontist. 2008 Jan;78(1):101-6.	
Nienkemper 2014	Nienkemper M, Wilmes B, Pauls A, Yamaguchi S, Ludwig B, Drescher D. Treatment efficiency of mini-implant-borne distalization depending on age and second-molar eruption. Journal of Orofacial Orthopedics/Fortschritte der Kieferorthopädie. 2014 Mar 1;75(2):118-32.	Retrospective design
Park 2003	Park HS. Clinical study on success rate of microscrew implants for orthodontic anchorage. Korean J Orthod. 2003 Jun 1;33(3):151-6.	Retrospective design
Park 2012	Park HM, Kim BH, Yang IH, Baek SH. Preliminary three-dimensional analysis of tooth movement and arch dimension change of the maxillary dentition in Class II division 1 malocclusion treated with first premolar extraction: conventional anchorage vs. mini-implant anchorage. The Korean Journal of Orthodontics. 2012 Dec 1;42(6):280-90.	Retrospective design
Park 2006	Park HS, Jeong SH, Kwon OW. Factors affecting the clinical success of screw implants used as orthodontic anchorage. American Journal of Orthodontics and Dentofacial Orthopedics. 2006 Jul 31;130(1):18-25.	Retrospective design
Sa'aed 2014	Sa'aed NL, Park CO, Bayome M, Park JH, Kim Y, Kook YA. Skeletal and dental effects of molar distalization using a modified palatal anchorage plate in adolescents. The Angle Orthodontist. 2014 Sep 5;85(4):657-64.	Retrospective design
Scheffler 2014	Scheffler NR, Proffit WR, Phillips C. Outcomes and stability in patients with anterior open bite and long anterior face height treated with temporary anchorage devices and a maxillary intrusion splint. American Journal of Orthodontics and Dentofacial Orthopedics. 2014 Nov 30;146(5):594-602.	Retrospective design
Scott Conley 2006	Scott Conley R, Jernigan C. Soft tissue changes after upper premolar extraction in Class II camouflage therapy. The Angle orthodontist. 2006 Jan;76(1):59-65.	Retrospective design
Sharma 2011	Sharma P, Valiathan A, Sivakumar A. Success rate of microimplants in a university orthodontic clinic. ISRN surgery. 2011 Apr 26;2011.	Retrospective design
Son 2015	Son S, Kim SS, Son WS, Kim YI, Kim YD, Shin SH. Miniscrews versus surgical archwires for intermaxillary fixation in adults after orthognathic surgery. The Korean Journal of Orthodontics. 2015 Jan 1;45(1):3-12.	Retrospective design
Suzuki 2013	Suzuki EY, Suzuki B. Maxillary molar distalization with the indirect Palatal miniscrew for Anchorage and Distalization Appliance (iPANDA). ORTHODONTICS: The Art & Practice of Dentofacial Enhancement. 2013 Mar 1;14(1).	Retrospective design
Takaki 2010	Takaki T, Tamura N, Yamamoto M, Takano N, Shibahara T, Yasumura T, Nishii Y, Sueishi K. Clinical study of temporary anchorage devices for orthodontic treatment. The Bulletin of	Retrospective design

	Tokyo Dental College. 2010;51(3):151-63.	
Topouzelis 2012	Topouzelis N, Tsaousoglou P. Clinical factors correlated with the success rate of miniscrews in orthodontic treatment. International journal of oral science. 2012 Apr 1;4(1):38-44.	Retrospective design
Tseng 2006	Tseng YC, Hsieh CH, Chen CH, Shen YS, Huang IY, Chen CM. The application of mini-implants for orthodontic anchorage. International journal of oral and maxillofacial surgery. 2006 Aug 31;35(8):704-7.	Retrospective design
Tsaousidis 2008	Tsaousidis G. Influence of insertion site on the failure rates of orthodontic miniscrews. Journal of Orofacial Orthopedics/ Fortschritte der Kieferorthopädie. 2008 Sep 1;69(5):349-56.	Retrospective design
Uribe 2015	Uribe F, Mehr R, Mathur A, Janakiraman N, Allareddy V. Failure rates of mini-implants placed in the infrazygomatic region. Progress in orthodontics. 2015 Sep 15;16(1):31.	Retrospective design
Xun 2013	Xun CL, Zhao H, Zeng XL, Wang X. Intrusion of overerupted maxillary molars with miniscrew implant anchorage: A radiographic evaluation. Journal of Huazhong University of Science and Technology [Medical Sciences]. 2013 Oct 1;33(5):780-5.	Retrospective design
Yao 2015	Yao CC, Chang HH, Chang JZ, Lai HH, Lu SC, Chen YJ. Revisiting the stability of mini-implants used for orthodontic anchorage. Journal of the Formosan Medical Association. 2015 Nov 30;114(11):1122-8.	Retrospective design
Yao 2005	Yao CC, Lee JJ, Chen HY, Chang ZC, Chang HF, Chen YJ. Maxillary molar intrusion with fixed appliances and mini-implant anchorage studied in three dimensions. The Angle orthodontist. 2005 Sep;75(5):754-60.	Retrospective design
Yi 2015	Yi LS, Mimi Y, Ming TC, Kelvin WC, Hung CW. A study of success rate of miniscrew implants as temporary anchorage devices in singapore. International journal of dentistry. 2015;2015:294670.	Retrospective design
Yilmaz 2015	Yılmaz A, Arman-Özçırpıcı A, Erken S, Polat-Özsoy Ö. Comparison of short-term effects of mini-implant-supported maxillary expansion appliance with two conventional expansion protocols. The European Journal of Orthodontics. 2015 Oct 1;37(5):556-64.	Retrospective design
Vasser 2015	Vassar JW, Karydis A, Trojan T, Fisher J. Dentoskeletal effects of a temporary skeletal anchorage device-supported rapid maxillary expansion appliance (TSADRME): A pilot study. The Angle Orthodontist. 2015 May 20;86(2):241-9.	Retrospective design
Wang 2008	Wang YC, Liou EJ. Comparison of the loading behavior of self-drilling and predrilled miniscrews throughout orthodontic loading. American Journal of Orthodontics and Dentofacial Orthopedics. 2008 Jan 31;133(1):38-43.	Retrospective design
Wang 2010	Wang Z, Zhang D, Liu Y, Zhao Z. Factors Associated with Buccal Mucosal Lesions Caused by the Interradicular	Retrospective design

	Miniscrew: A Preliminary Report. International Journal of Oral & Maxillofacial Implants. 2010 Dec 1;25(6).	
Wang 2012	Wang Y, He D, Yang C, Wang B, Qian W. An easy way to apply orthodontic extraction for impacted lower third molar compressing to the inferior alveolar nerve. Journal of Cranio-Maxillofacial Surgery. 2012 Apr 30;40(3):234-7.	Retrospective design
Wang 2011	Wang Q, Jia P, Anderson NK, Wang L, Lin J. Changes of pharyngeal airway size and hyoid bone position following orthodontic treatment of Class I bimaxillary protrusion. The Angle Orthodontist. 2011 Jul 27;82(1):115-21.	Retrospective design
Wu 2009	Wu TY, Kuang SH, Wu CH. Factors associated with the stability of mini-implants for orthodontic anchorage: a study of 414 samples in Taiwan. Journal of Oral and Maxillofacial Surgery. 2009 Aug 31;67(8):1595-9.	Retrospective design
Wilmes 2010	Wilmes B, Nienkemper M, Drescher D. Application and effectiveness of a mini-implant-and tooth-borne rapid palatal expansion device: the hybrid hyrax. World J Orthod. 2010 Dec 1;11(4):323-0.	Retrospective design
Wilmes 2010	Wilmes B, Drescher D. Application and effectiveness of the Beneslider: a device to move molars distally. World J Orthod. 2010 Dec 1;11(4):331-40.	Retrospective design

Search strategies and hits in electronic databases

Electronic Databases	Conducted search strategy (20th October 2017)	Search Hits
Cochrane central register of controlled trials	ID Search Hits<br>#1 MeSH descriptor: [Orthodontic Anchorage Procedures] explode all trees<br>#2 (Miniscrew* or "Mini screw*" or Mini-screw* or Mini-implant* or "Mini implant*" or "Temporary anchorage" or "Temporary anchorage device*" or TADs or TAD or "skeletal anchorage" or "bone anchorage")<br>#3 Ortho*<br>#4 (#1 or #2) and #3	114<br>330<br>28679<br>163
Scopus	(TITLE-ABS-KEY ((miniscrew* OR "Mini screw*" OR mini-screw* OR mini-implant* OR "Mini implant*" OR "Temporary anchorage" OR "Temporary anchorage device*" OR tads OR tad OR "skeletal anchorage" OR "bone anchorage")) AND TITLE-ABS-KEY (ortho*))<br>Search: Title/Abstract/keywords	1850
Web of Knowledge	TOPIC: (Ortho*) AND TOPIC: ((Miniscrew* OR "Mini screw*" OR Mini-screw* OR Mini-implant* OR "Mini implant*" OR "Temporary anchorage" OR "Temporary anchorage device*" OR TADs OR TAD OR "skeletal anchorage" OR "bone anchorage"))<br>Timespan: All years. Indexes: SCI-EXPANDED, SSCI, A&HCI, CPCI-S, CPCI-SSH, BKCI-S, BKCI-SSH, ESCI, CCR-EXPANDED, IC.	1242
Medline via EBSCOhost	(MH "Orthodontic Anchorage Procedures")<br>(Miniscrew* OR "Mini screw*" OR Mini-screw* OR Mini-implant* OR "Mini implant*" OR "Temporary anchorage" OR "Temporary anchorage device*" OR TADs OR TAD OR "skeletal anchorage" OR "bone anchorage")<br>S1 OR S2<br>Ortho*<br>(Ortho*) AND (S3)	1786<br>3651<br>4365<br>568,596<br>2427
PubMed	(("Orthodontic Anchorage Procedures"[Mesh]) OR ((Miniscrew* OR "Mini screw*" OR Mini-screw* OR Mini-implant* OR "Mini implant*" OR "Temporary anchorage" OR "Temporary anchorage device*" OR TADs OR TAD OR "skeletal anchorage" OR "bone anchorage"))) AND Orthodon*	2279
