# Supplementary material for: Role of anatomical sites and correlated risk factors on the survival of orthodontic miniscrew implants: a systematic review and meta-analysis
Source: Prog Orthod. 2018 Sep 24;19:36. doi: 10.1186/s40510-018-0225-1 (PMC6151309; doi:10.1186/s40510-018-0225-1)
Supplement: Supplementary file 3 — Table S2. List of communications. (PDF 137 kb) [file 40510_2018_225_MOESM3_ESM.pdf]

**List of communications**

| Study | Reason for inquiry | Person/ inquiry date | Status |
|---|---|---|---|
| Falkensammer F, Rausch-Fan X, Arnhart C, Krall C, Schaden W, Freudenthaler J. Impact of extracorporeal shock-wave therapy on the stability of temporary anchorage devices in adults: A single-center, randomized, placebo-controlled clinical trial. American Journal of Orthodontics and Dentofacial Orthopedics. 2014 Oct 1;146(4):413-22. | Lack of clarity regarding the miniscrew insertion sites | Dr Falkensammer 5-10-2016 | Response pending |
| -Wilmes B, Drescher D. Application and effectiveness of the Beneslider: a device to move molars distally. World J Orthod. 2010 Dec 1;11(4):331-40.<br>-Wilmes B, Nienkemper M, Drescher D. Application and effectiveness of a mini-implant-and tooth-borne rapid palatal expansion device: the hybrid hyrax. World J Orthod. 2010 Dec 1;11(4):323-0.<br>-Nienkemper M, Wilmes B, Pauls A, Drescher D. Maxillary protraction using a hybrid hyrax-facemask combination. Progress in orthodontics. 2013 Dec;14(1):5. | Study design | Dr Wilmes 7-2-2016 | Response pending |
| Suzuki EY, Suzuki B. Maxillary molar distalization with the indirect Palatal miniscrew for Anchorage and Distalization Appliance (iPANDA). ORTHODONTICS: The Art & Practice of Dentofacial Enhancement. 2013 Mar 1;14(1). | Study design | Dr Suzuki 7-2-2016 | Response pending |
| Gelgor IE, Karaman AI, Buyukyilmaz T. Comparison of 2 distalization systems supported by intraosseous screws. American Journal of Orthodontics and Dentofacial Orthopedics. 2007 Feb 1;131(2):161-e1. | Study design | Dr Gelgor 7-2-2016 | Response pending |
| Virang B, Makhija PG, Belludi A, Bhatia V, Padmawar SS, Gupta A. Evaluation of Titanium Miniscrew Implants as a Source of Intraoral Anchorage for en masse Intrusion of Maxillary Anterior Teeth: A Clinical Study. Journal of Indian Orthodontic Society. 2013 Oct 1;47(4):184. | Clarification about OMIs that failed | Dr Virang 7-10-2016 | Response pending |
| Kayalar E, Schauseil M, Kuvat SV, Emekli U, Fıratlı S. Comparison of tooth-borne and hybrid devices in surgically assisted rapid maxillary expansion: a randomized clinical cone-beam computed tomography study. Journal of Cranio-Maxillo-Facial Surgery. 2016 Mar 1;44(3):285-93. | Clarification about OMI failure and blinding methods | Dr kayalar 7-11-2016 | Response received |
| -Aboul SM, El-Beialy AR, El-Sayed KM, Selim EM, El-Mangoury NH, Mostafa | Clarification about | Dr Mangoury | Response |

| | | | |
|---|---|---|---|
| YA. Miniscrew implant-supported maxillary canine retraction with and without corticotomy-facilitated orthodontics. American Journal of Orthodontics and Dentofacial Orthopedics. 2011 Feb 1;139(2):252-9.<br>- El-Dawlatly MM, Abou-EL-Ezz AM, El-Sharaby FA, Mostafa YA. Zygomatic mini-implant for Class II correction in growing patients. Journal of Orofacial Orthopedics/Fortschritte der Kieferorthopädie. 2014 May 1;75(3):213-25. | randomisation process | 8-6-2016 | pending |
| Aras I, Tuncer AV. Comparison of anterior and posterior mini-implant-assisted maxillary incisor intrusion: Root resorption and treatment efficiency. The Angle Orthodontist. 2016 Jan 7;86(5):746-52. | Clarification about randomisation process | Dr Aras<br>8-6-2016 | Response received |
| Aslan BI, Kucukkaraca E, Turkoz C, Dincer M. Treatment effects of the Forsus Fatigue Resistant Device used with miniscrew anchorage. The Angle Orthodontist. 2013 Jun 17;84(1):76-87. | Clarification about randomisation process | Dr Aslan<br>8-6-2016 | Response pending |
| Bechtold TE, Kim JW, Choi TH, Park YC, Lee KJ. Distalization pattern of the maxillary arch depending on the number of orthodontic miniscrews. The Angle Orthodontist. 2012 Sep 12;83(2):266-73. | Clarification about randomisation process | Dr Bechtold<br>8-6-2016 | Response pending |
| Davoody AR, Posada L, Utreja A, Janakiraman N, Neace WP, Uribe F, Nanda R. A prospective comparative study between differential moments and miniscrews in anchorage control. European journal of orthodontics. 2012 Aug 16;35(5):568-76. | Clarification about allocation concealment | Dr Uribe<br>8-6-2016 | Response pending |
| Ge YS, Liu J, Chen L, Han JL, Guo X. Dentofacial effects of two facemask therapies for maxillary protraction: Miniscrew implants versus rapid maxillary expanders. The Angle Orthodontist. 2012 May 28;82(6):1083-91. | Clarification about randomisation process | Dr Ge<br>8-6-2016 | Response pending |
| -Iwai H, Motoyoshi M, Uchida Y, Matsuoka M, Shimizu N. Effects of tooth root contact on the stability of orthodontic anchor screws in the maxilla: comparison between self-drilling and self-tapping methods. American Journal of Orthodontics and Dentofacial Orthopedics. 2015 Apr 1;147(4):483-91.<br>-Son S, Motoyoshi M, Uchida Y, Shimizu N. Comparative study of the primary stability of self-drilling and self-tapping orthodontic miniscrews. American Journal of Orthodontics and Dentofacial Orthopedics. 2014 Apr 1;145(4):480-5. | Clarification about randomisation process | Dr Motoyoshi<br>8-6-2016 | Response pending |
| Lehnen S, McDonald F, Bourauel C, Jäger A, Baxmann M. Expectations, acceptance and preferences of patients in treatment with orthodontic mini-implants. Journal of Orofacial Orthopedics/Fortschritte der Kieferorthopädie. 2011 Jul 1;72(3):214-22. | Clarification about allocation concealment | Dr Lehnen<br>8-6-2016 | Response pending |

| | | | |
|---|---|---|---|
| Liu YH, Ding WH, Liu J, Li Q. Comparison of the differences in cephalometric parameters after active orthodontic treatment applying mini-screw implants or transpalatal arches in adult patients with bialveolar dental protrusion. Journal of oral rehabilitation. 2009 Sep 1;36(9):687-95. | Clarification about randomisation process | Dr Liu 8-6-2016 | Response pending |
| Sarul M, Minch L, Park HS, Antoszewska-Smith J. Effect of the length of orthodontic mini-screw implants on their long-term stability: a prospective study. The Angle Orthodontist. 2014 Apr 21;85(1):33-8. | Clarification about randomisation process | Dr Sarul 8-6-2016 | Response received |
| Suzuki M, Deguchi T, Watanabe H, Seiryu M, Iikubo M, Sasano T, Fujiyama K, Takano-Yamamoto T. Evaluation of optimal length and insertion torque for miniscrews. American Journal of Orthodontics and Dentofacial Orthopedics. 2013 Aug 1;144(2):251-9. | Clarification about randomisation process | Dr Suzuki 8-6-2016 | Response pending |
| Toklu MG, Germec-Cakan D, Tozlu M. Periodontal, dentoalveolar, and skeletal effects of tooth-borne and tooth-bone-borne expansion appliances. American Journal of Orthodontics and Dentofacial Orthopedics. 2015 Jul 1;148(1):97-109. | Clarification about randomisation process | Dr Cakan 8-6-2016 | Partial response was received regarding OMI failure. Randomisation process could not be clarified |
| Türköz Ç, Ataç MS, Tuncer C, Baloş Tuncer B, Kaan E. The effect of drill-free and drilling methods on the stability of mini-implants under early orthodontic loading in adolescent patients. The European Journal of Orthodontics. 2010 Dec 3;33(5):533-6. | Clarification about randomisation process | Dr Türköz 8-6-2016 | Response pending |
