# Supplementary material for: Role of anatomical sites and correlated risk factors on the survival of orthodontic miniscrew implants: a systematic review and meta-analysis
Source: Prog Orthod. 2018 Sep 24;19:36. doi: 10.1186/s40510-018-0225-1 (PMC6151309; doi:10.1186/s40510-018-0225-1)
Supplement: Supplementary file 4 — Table S3. Additional risk factors. (PDF 168 kb) [file 40510_2018_225_MOESM4_ESM.pdf]

Additional risk factors

Study	No. of failed OMIs perforating sinus/total	No. of failed OMIs without sinus perforation/total	No. of failed OMIs with root contact/total	No. of failed OMIs without root contact/total	Cortical bone thickness outcomes	Method of measurement
El beialy 2009	-	-	(7/12)	(0/28)	-	CT scan
Iwai 2015	-	-	(6/29)	(4/113)	Self-drilling mean of 1.02 mm and self-tapping mean of 1.05 mm	CBCT
Janson 2012*	-	-	(3/20)*	(1/20)**	-	Bitewing
Kim 2010	(1/4)	(1/46)	(1/15)	(1/35)	-	CBCT
Miyazawa 2010	-	-	(0/0)	(4/44)	-	CBCT
Motoyoshi 2009	-	-	-	-	≥ 1.0 mm (153 total OMIs/ 13 failed) <1.0mm (56 total OMIs /11 failed)	CT scan
Motoyoshi 2015	(1/8)	(4/74)	-	-	-	CBCT
Motoyoshi 2016	-	-	(7/37)	(2/165)	Successful OMIs with a mean of 0.81 mm (SD 0.32). Failed OMIs with a mean of 0.79 mm (SD 0.39)	CBCT
Samrit 2012	-	-	-	-	Cortical bone (34 successful with median HU 975.3 and 4 failed with median HU 1157.45) Cancellous bone (34 successful OMIs with median HU 501 and 4 failed OMIs with median HU 632.45)	CT scan
Shigeeda 2014	-	-	(6/35)	(2/130)	-	CBCT
Suzuki 2013	(0/0)	(8/122)	(0/0)	(27/186)	Maxilla (success mean of 993.3 HU and for the failed was 1000.6 HU) Mandible (success mean was 902.8 HU and failed mean was 946.7 HU)	CBCT
Watanabe	-	-	(10/17)	(6/111)	Maxilla: (112 successful with mean	CBCT

2012					1.63 mm and 10 failed with mean 1.53 mm)<br>Mandible (38 successful with a mean of 2.34 mm and 14 failed with a mean of 2.19 mm)	
Watanabe 2017	-	-	-	-	Success group 1.34 mm SD 0.35 and failure group 0.99 mm SD 0.09	CBCT

*Data presented with root proximity less than 3mm

**Data presented for OMIs away from the root by 3mm
