# Supplementary material for: Role of anatomical sites and correlated risk factors on the survival of orthodontic miniscrew implants: a systematic review and meta-analysis
Source: Prog Orthod. 2018 Sep 24;19:36. doi: 10.1186/s40510-018-0225-1 (PMC6151309; doi:10.1186/s40510-018-0225-1)
Supplement: Supplementary file 5 — Table S4. Grading of evidence. (PDF 848 kb) [file 40510_2018_225_MOESM5_ESM.pdf]

GRADE approach for OMI risk factors

Outcomes	Anticipated absolute effects* (95% CI)		Relative effect (95% CI)	№ of participants (studies)	Certainty of the evidence (GRADE)	Comments
	Risk with [comparison]	Risk with [intervention]				
OMI failure in the right versus left insertion sites assessed with: Total number of failure events	60 per 1,000	**94 per 1,000** (63 to 140)	**RR 1.57** (1.05 to 2.35)	1008 OMIs (14 studies)	⨁◯◯◯ VERY LOW [a]	Risk with [intervention] denotes the anticipated risks for failure when OMIs are inserted in the right side
OMI failure when the root is in contact versus when the root is not in contact assessed with: Total number of failure events	57 per 1,000	**493 per 1,000** (291 to 837)	**RR 8.7** (5.13 to 14.77)	957 OMIs (8 studies)	⨁⨁⨁◯ MODERATE [a]	Risk with [intervention] denotes the anticipated risks for failure when OMIs hit the root. Evidence was upgraded two levels due to large effect.
OMI failure when the maxillary sinus is perforated versus when the maxillary sinus isn't perforated assessed with: Total number of failure events	54 per 1,000	**283 per 1,000** (79 to 1,000)	**RR 5.26** (1.47 to 18.74)	254 OMIs (3 studies)	⨁◯◯◯ VERY LOW [a,b]	Risk with [intervention] denotes the anticipated risks for failure when OMIs perforate the maxillary sinus

***The risk in the intervention group** (and its 95% confidence interval) is based on the assumed risk in the comparison group and the **relative effect** of the intervention (and its 95% CI).

CI: Confidence interval; **RR:** Risk ratio, **OMI:** Orthodontic miniscrew implant

GRADE Working Group grades of evidence
High certainty: We are very confident that the true effect lies close to that of the estimate of the effect
Moderate certainty: We are moderately confident in the effect estimate: The true effect is likely to be close to the estimate of the effect, but there is a possibility that it is substantially different
Low certainty: Our confidence in the effect estimate is limited: The true effect may be substantially different from the estimate of the effect
Very low certainty: We have very little confidence in the effect estimate: The true effect is likely to be substantially different from the estimate of effect

Explanations

a. Downgraded one level for risk of bias within some of the included studies

b. Downgraded one level for wide confidence intervals

Summary of findings for insertion sites

Outcomes	Certainty assessment							Summary of findings	Certainty
	№ of studies	Study design	Risk of bias	Inconsistency	Indirectness	Imprecision	Other considerations		
OMI failure in palatal insertion sites	14	RCTs + observational studies	serious [a]	not serious	not serious	not serious	strong association	A total number of 608 OMIs were inserted in three distinct palatal insertion sites with a pooled failure rate of 4.7% (95% Cl 2.7-8.1).	⨁⨁◯◯ LOW
OMI failure in maxillary buccal insertion sites	43	RCTs + observational studies	serious [a]	serious [b]	not serious	not serious	publication bias strongly suspected strong association [c]	A total number of 2637 OMIs were inserted in three distinct maxillary buccal insertion sites with a pooled failure rate of 9.6% (95% Cl 7.6-12.1).	⨁◯◯◯ VERY LOW
OMI failure in mandibular buccal insertion sites	11	RCTs + observational studies	serious [a]	serious [d]	not serious	not serious	none	A total number of 550 OMIs were inserted in two distinct mandibular insertion sites with a pooled failure rate of 12.3% (95% Cl 7.3-20.1).	⨁◯◯◯ VERY LOW

Explanations

a. Downgraded one level for risk of bias within some of the included studies
b. Downgraded one level for statistical heterogeneity
c. Downgraded one level for suspicion of publication bias
d. Downgraded one level for statistical heterogeneity and gap differences between reported failure rates in the studies
