# Supplementary material for: Role of anatomical sites and correlated risk factors on the survival of orthodontic miniscrew implants: a systematic review and meta-analysis
Source: Prog Orthod. 2018 Sep 24;19:36. doi: 10.1186/s40510-018-0225-1 (PMC6151309; doi:10.1186/s40510-018-0225-1)
Supplement: Supplementary file 6 — Figure S1. Sensitivity analyses. (PDF 530 kb) [file 40510_2018_225_MOESM6_ESM.pdf]

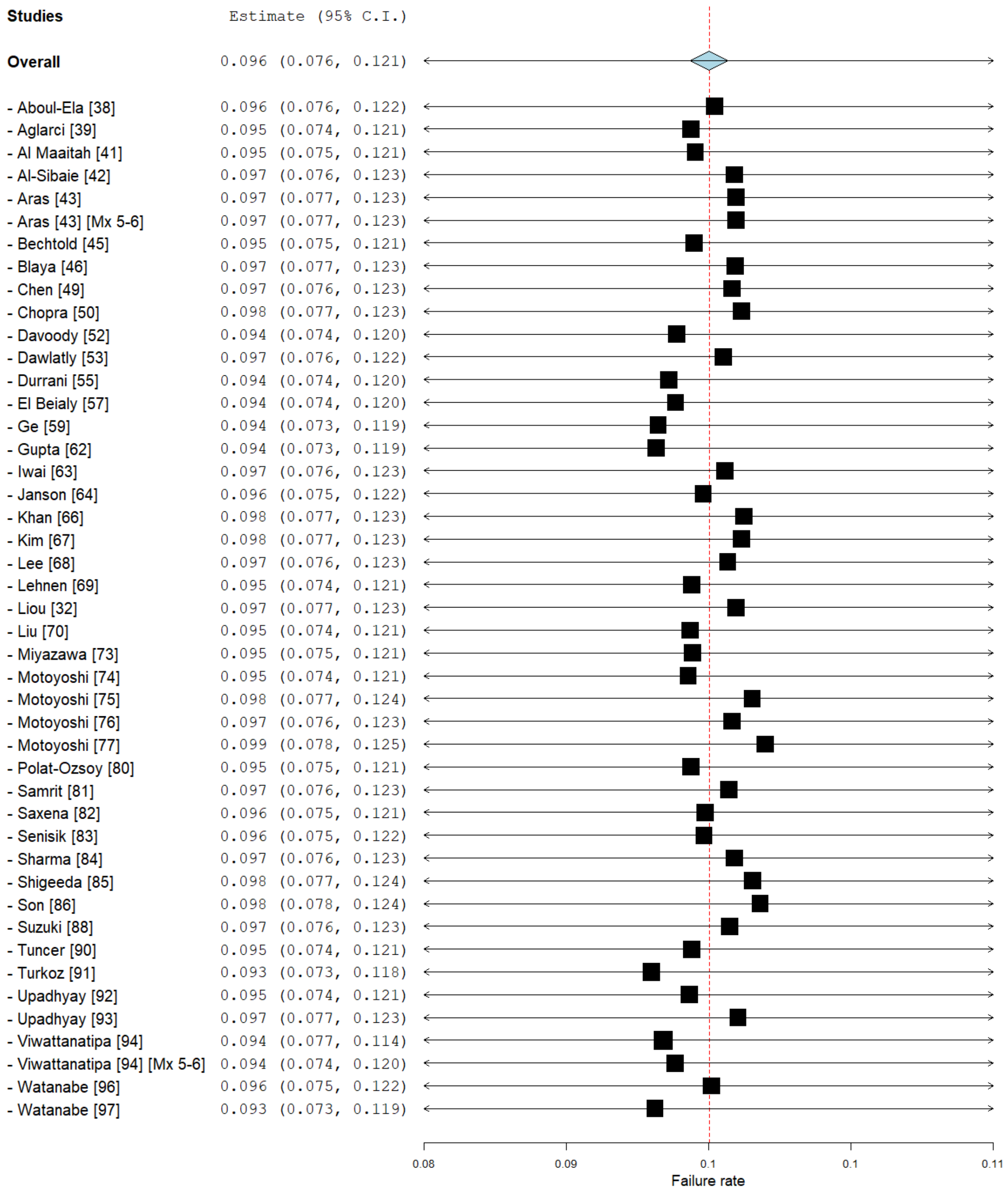

(a)

(a) Sensitivity test for maxillary buccal insertion sites with one study removed

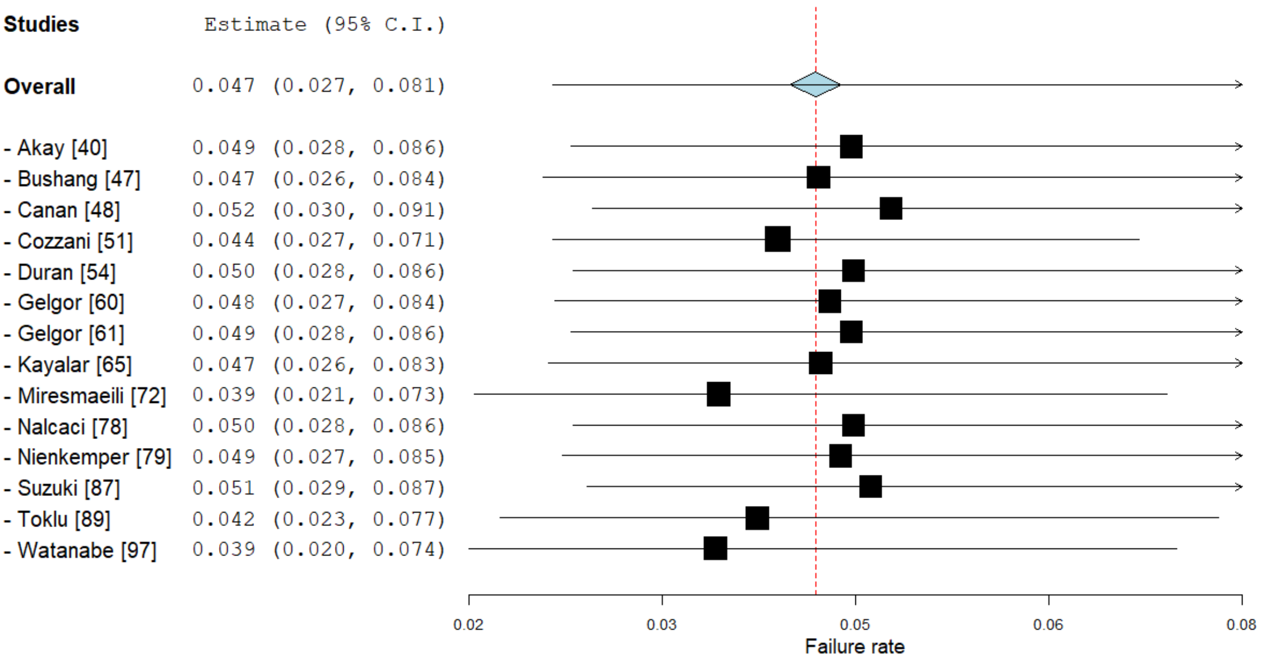

(b)

(b) Sensitivity test for palatal insertion sites with one study removed

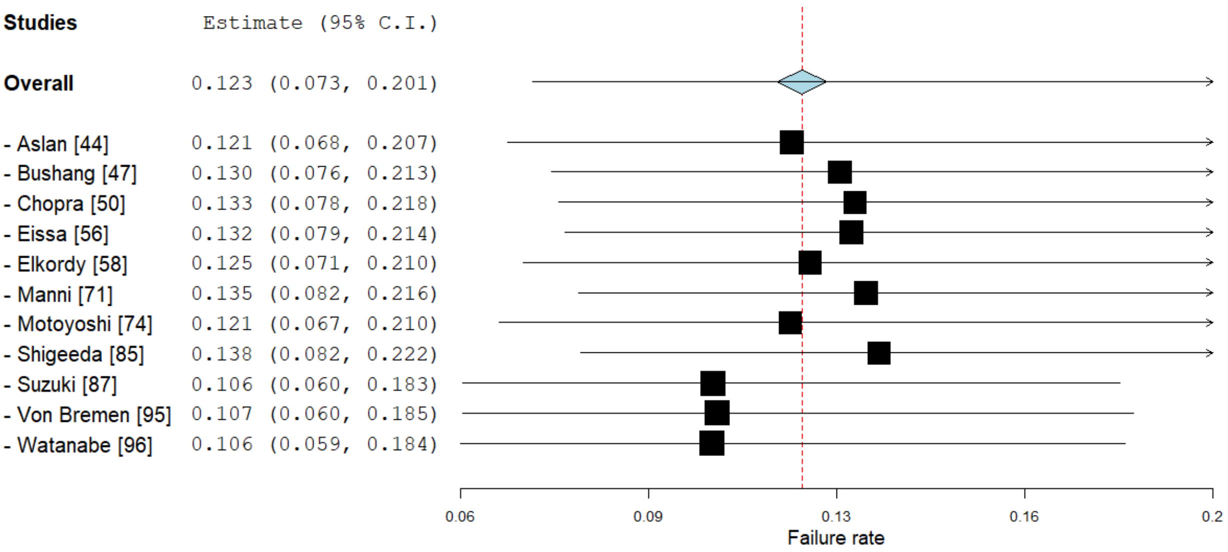

(c)

(c) Sensitivity test for mandibular insertion sites with one study removed

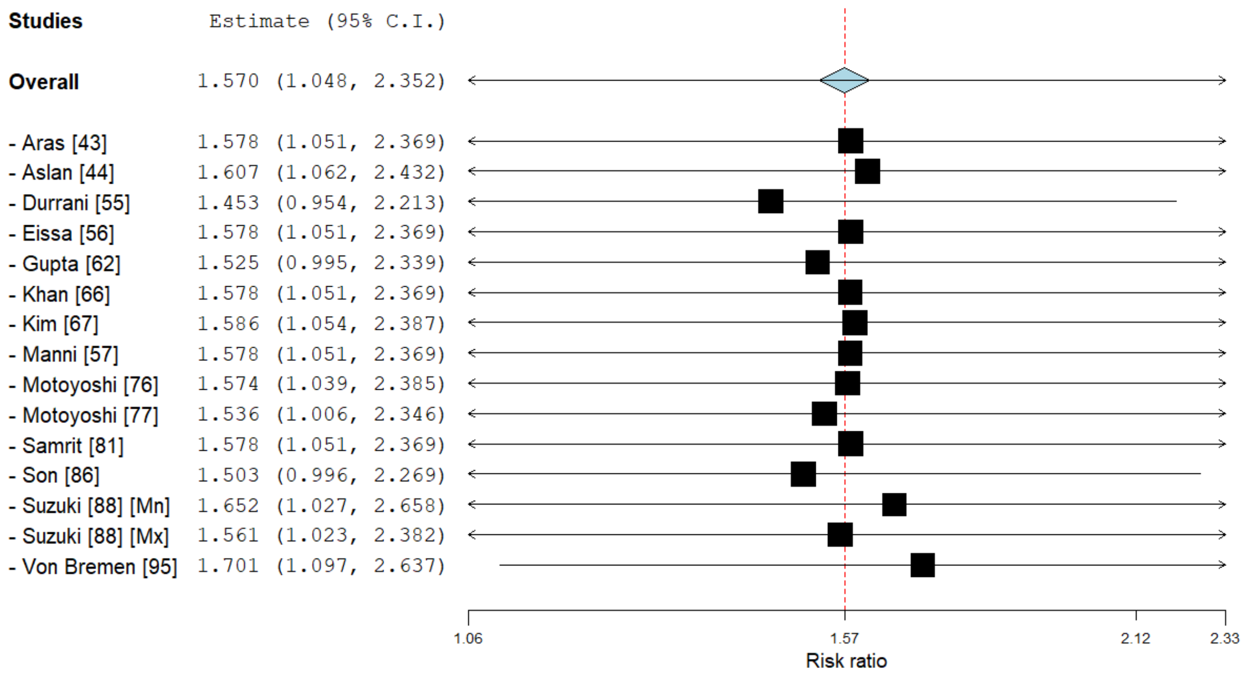

(d)

(d) Sensitivity test for insertion side (right or left) risk factor with one study removed

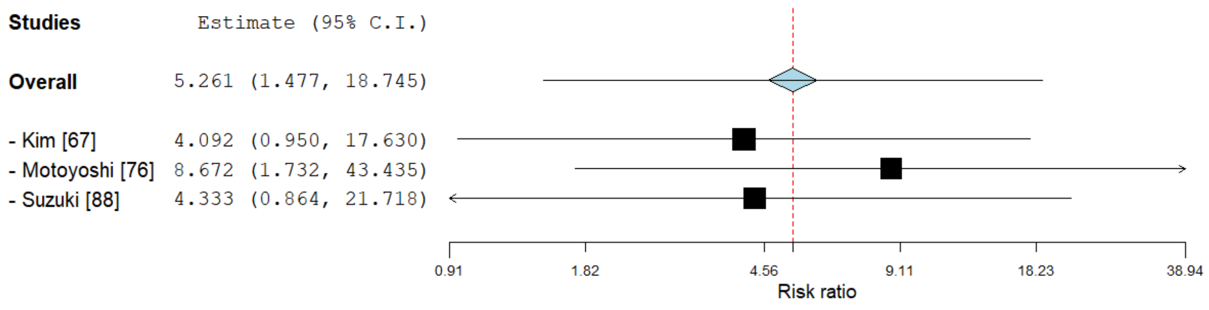

(e)

(e) Sensitivity test for the maxillary sinus risk factor with one study removed

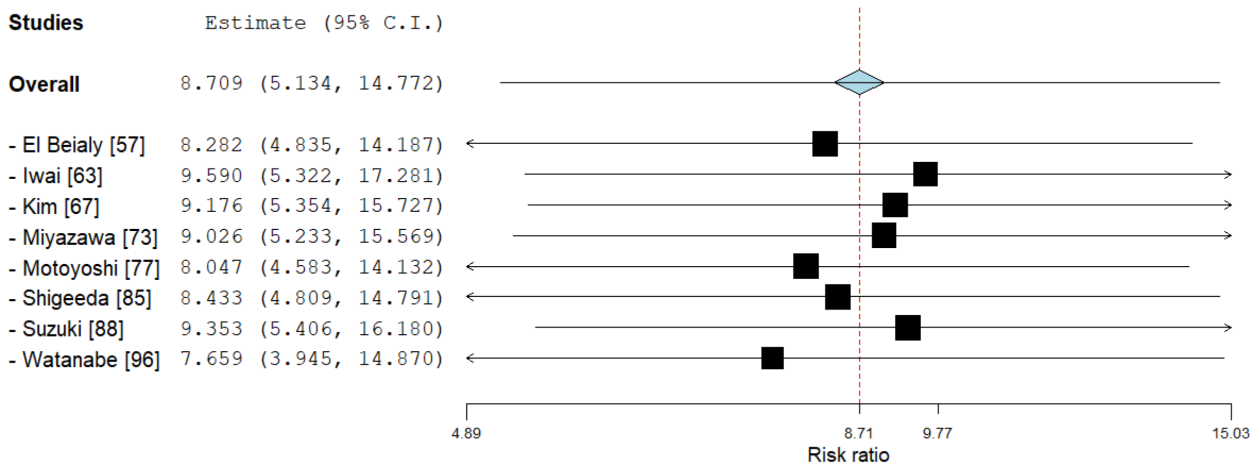

(f)

(f) Sensitivity test for the root contact risk factor with one study removed

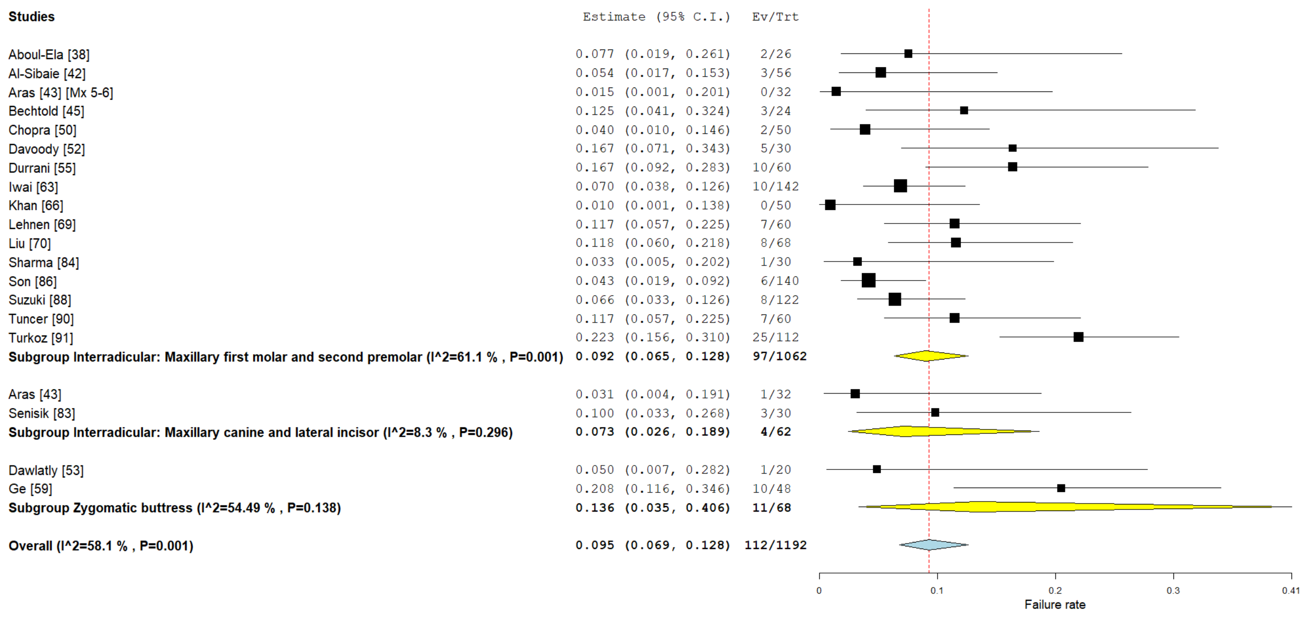

(g)

(g) Sensitivity test for maxillary buccal insertion sites with non-randomised trials removed

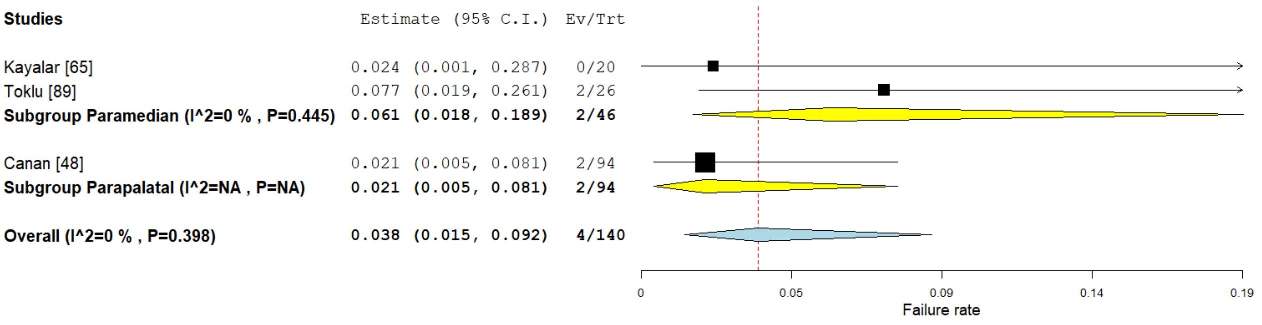

(h)

(h) Sensitivity test for palatal insertion sites with non-randomised trials removed

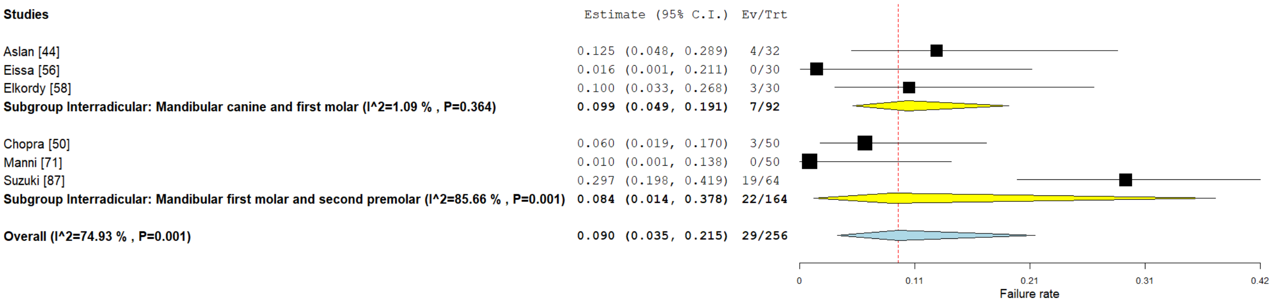

(i)

(i) Sensitivity test for mandibular insertion sites with non-randomised trials removed

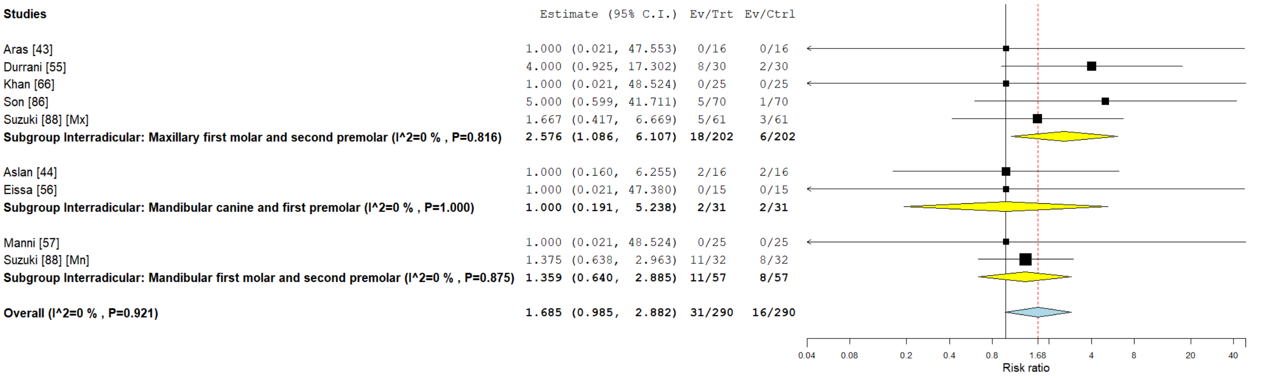

(j)

(j) Sensitivity test for insertion side (right or left) risk factor with non-randomised trials removed

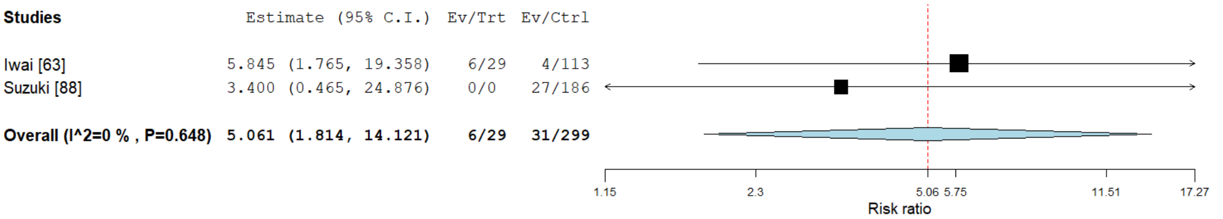

(k)

(k) Sensitivity test for the root contact risk factor with non-randomised trials removed

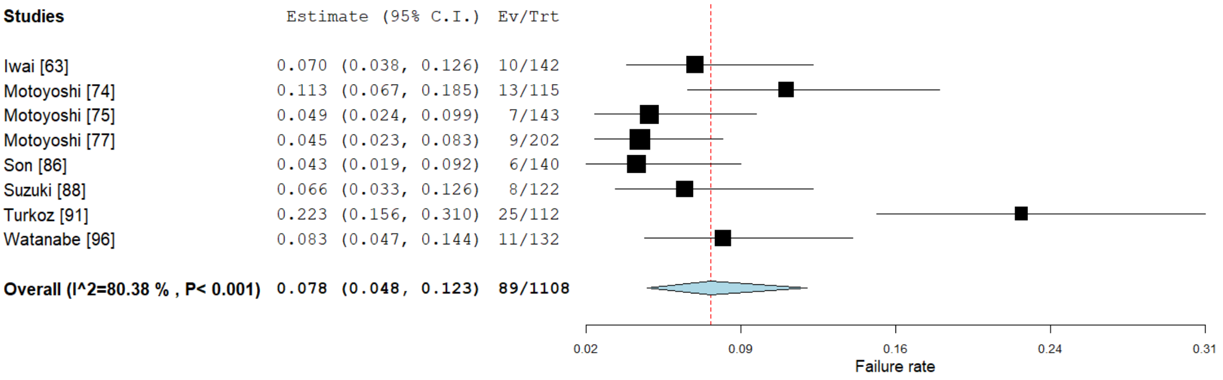

(L) Sensitivity test for OMIs inserted in maxillary buccal locations by excluding small studies (< 100 OMIs)

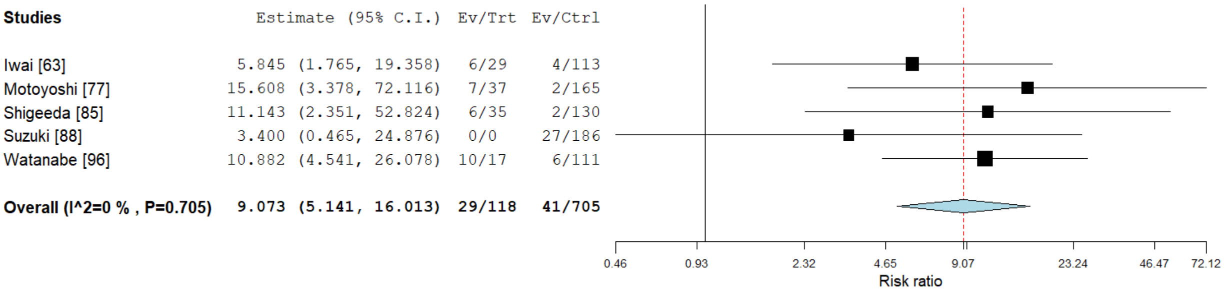

(M) Sensitivity test for the root contact risk factor with small studies excluded (< 100 OMIs)

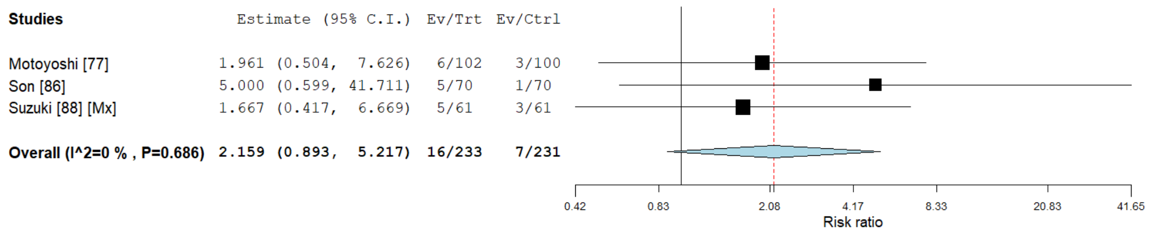

(N) Sensitivity test for the side of insertion risk factor with small studies excluded (< 100 OMIs)
