# Supplementary material for: Role of anatomical sites and correlated risk factors on the survival of orthodontic miniscrew implants: a systematic review and meta-analysis
Source: Prog Orthod. 2018 Sep 24;19:36. doi: 10.1186/s40510-018-0225-1 (PMC6151309; doi:10.1186/s40510-018-0225-1)
Supplement: Supplementary file 7 — Table S5. Statistical summary. (PDF 996 kb) [file 40510_2018_225_MOESM7_ESM.pdf]

Statistical summary for reported insertion sites

	Random-effects				Heterogeneity				
Insertion sites	Number of studies	Event rate (%)	95% prediction interval	95% CI	I^2 (95% CI)	H (95% CI)	Tau^2	Q (df)	P-value
**Maxillary buccal insertion sites**	43*	9.6%	2.5-30.3	7.6-12.1	68.9% (58-77)	1.8 (1.5-2.1)	0.47	141.485 (44)	< 0.001
Subgroup Interradicular: Maxillary first molar and second premolar	37	9.2%	3.4-22.4	7.4-11.4	55.6% (35-69)	1.5 (1.2-1.8)	0.25	81.099 (36)	< 0.001
Subgroup Interradicular: Maxillary canine and lateral incisor	4	9.7%	2.3-32.7	5.1-17.6	0% (0-85)	1 (1-2.6)	0	1.791 (3)	0.617
Subgroup: Zygomatic buttress	4	16.4%	0.06-98.2	4.9-42.5	83.7% (59-94)	2.5 (1.6-3.9)	1.28	18.412 (3)	< 0.001
**Mandibular buccal insertion sites**	11	12.3%	2-48.3	7.3-20.1	75% (55-86)	2 (1.5-2.7)	0.61	40.004 (10)	< 0.001
Subgroup Interradicular: Mandibular first molar and second premolar	8	13.5%	6.2-26.7	7.3-23.6	79.7% (61-90)	2.2 (1.6-3.1)	0.66	34.568 (7)	< 0.001
Subgroup Interradicular: Mandibular canine and first molar	3	9.9%	0.07-94.4	4.9-19.1	1.09% (0-90)	1 (1-3.1)	0.006	2.022 (2)	0.364
**Palatal insertion sites**	14	4.7%	1-18	2.7-8.1	37.5% (0-67)	1.3 (1-1.7)	0.38	20.820 (13)	0.077
Subgroup: Paramedian	6	4.8%	0.2-50	1.6-13.4	48.1% (0-79)	1.4 (1-2.2)	0.84	9.647 (5)	0.086
Subgroup: Parapalatal	5	5.5%	0.9-27.4	2.8-10.7	33.2% (0-75)	1.2 (1-2)	0.21	5.993 (4)	0.200
Subgroup: Midpalatal	3	1.3%	0-99.7	0.3-6	0% (0-90)	1 (1-3.1)	0	0.164 (2)	0.921

*Studies by Aras [43] and Viwattanatipa [94] reported two independent sites in this forest plot

Statistical summary for reported insertion sites (non-randomised studies removed)

		Random-effects			Heterogeneity				
Exclusion of non-randomized studies	Number of studies	Event rate (%)	95% prediction interval	95% CI	I^2 (95% CI)	H (95% CI)	Tau^2	Q (df)	P-value
**Maxillary buccal insertion sites**	19*	9.5%	3-25.8	6.9-12.8	58.1% (31-74)	1.5 (1.2-2)	0.29	45.347 (19)	< 0.001
Subgroup Interradicular: Maxillary first molar and second premolar	16	9.2%	2.7-26.3	6.5-12.8	61.1% (33-77)	1.6 (1.2-2.1)	0.31	38.557 (15)	< 0.001
Subgroup Interradicular: Maxillary first molar and second premolar	2	7.3%	-	2.6-18.9	8.3%	-	0.06	1.091 (1)	0.296
Subgroup: Zygomatic buttress	2	13.6%	-	3.5-40.6	54.4%	-	0.7	2.197 (1)	0.138
**Mandibular buccal insertion sites**	6	9%	4.2-69.8	3.5-21.5	74.9% (43-89)	2 (1.3-3)	1.02	19.947 (5)	0.001
Subgroup Interradicular: Mandibular first molar and second premolar	3	8.4%	0-100	1.4-37.8	85.6% (58-95)	2.6 (1.5-4.5)	2.17	13.942 (2)	< 0.001
Subgroup Interradicular: Mandibular canine and first molar	3	9.9%	0.07-94.4	4.9-19.1	1.09% (0-90)	1 (1-3.1)	0.006	2.022 (2)	0.364
**Palatal insertion sites**	3	3.8%	0-94.7	1.5-9.2	0% (0-90)	1 (1-3.1)	0	1.840 (2)	0.398
Subgroup: Paramedian	2	6.1%	-	1.8-18.9	0%	-	0	0.583 (1)	0.445
Subgroup: Parapalatal	1	2.1%	-	0.5-8.1	-	-	-	-	-

*Study by Aras [43] reported two independent sites in this forest plot

Statistical summary for risk factors

	Random-effects				Heterogeneity				
Risk factors	Number of studies	Risk ratio	95% prediction interval	95% CI	I^2 (95% CI)	H (95% CI)	Tau^2	Q (df)	P-value
**Influence of root contact**	8	8.7	4.5-16.8	5.1-14.7	0% (0-68)	1 (1-1.8)	0	4.25 (7)	0.750
**Influence of side of insertion**	14*	1.57	1-2.4	1.05-2.35	0% (0-54)	1 (1-1.5)	0	4.407 (14)	0.992
Subgroup Interradicular: Mandibular first molar and second premolar	3**	1.22	0.02-64.5	0.66-2.25	0% (0-90)	1 (1-3.1)	0	0.243 (2)	0.885
Subgroup Interradicular: Mandibular canine and first molar	2	1	-	0.19-5.23	0%	-	0	0 (1)	1
Subgroup Interradicular: Maxillary first molar and second premolar	10	2	1-4	1.16-3.64	0% (0-62)	1 (1-1.6)	0	2.362 (9)	0.984
**Influence of maxillary sinus perforation**	3	5.26	0.0014-19878	1.47-18.74	0% (0-90)	1 (1-3.1)	0	1.053 (2)	0.591

*Study by Suzuki [88] reported two independent sites in this forest plot. Studies by Chopra [50] and Motoyoshi [74] reported mixed insertion sites for the side of insertion and were not included in this particular quantitative synthesis

** Study by Samrit [81] did not have adequate number of miniscrews to be included in the analysis of mandibular side of insertion failure rates. It was only considered for the maxillary first molar and second premolar side of insertion risk factor

Statistical summary for risk factors (non-randomised studies removed)

Exclusion of non-randomized studies	Number of studies	Risk ratio	95% prediction interval	95% CI	I^2 (95% CI)	H (95% CI)	Tau^2	Q (df)	P-value
	Random-effects				Heterogeneity				
**Influence of root contact**	2	5.06	-	1.81-14.12	0%	-	0	0.209 (1)	0.648
**Influence of side of insertion**	8*	1.68	0.9-3.2	0.98-2.88	0% (0-65)	1 (1-1.7)	0	3.209 (8)	0.921
Subgroup Interradicular: Mandibular first molar and second premolar	2	1.35	-	0.64-2.88	0%	-	0	0.025 (1)	0.875
Subgroup Interradicular: Mandibular canine and first molar	2	1	-	0.19-5.23	0%	-	0	0 (1)	1
Subgroup Interradicular: Maxillary first molar and second premolar	5	2.57	0.6-10.5	1.08-6.1	0% (0-79)	1 (1-2.2)	0	1.560 (4)	0.816

*Study by Suzuki [88] reported two independent sites in this forest plot

Statistical summary after excluding small studies

	Random-effects				Heterogeneity				
Exclusion of small studies (less than 100 OMIs)	Number of studies	Effect estimate	95% prediction interval	95% CI	I^2 (95% CI)	H (95% CI)	Tau^2	Q (df)	P-value
**Influence of root contact**	5	9.07 RR	3.6-22.8	5.14-16.01	0% (0-79)	1 (1-2.2)	0	2.168 (4)	0.7
**Influence of side of insertion (Interradicular: Maxillary first molar and second premolar)**	3	2.16 RR	0.008-586	0.893-5.217	0% (0-90)	1 (1-3.1)	0	0.755 (2)	0.68
**Interradicular: Maxillary first molar and second premolar**	8	7.8 ER	1.4-31.9	4.8-12.3	80.3% (62-90)	2.3 (1.6-3.1)	0.42	35.6 (7)	< 0.001

RR: risk ratio; ER: event rate (%)
